# New Insights Into the Evolution of Corticotropin-Releasing Hormone Family With a Special Focus on Teleosts

**DOI:** 10.3389/fendo.2022.937218

**Published:** 2022-07-22

**Authors:** Gersende Maugars, Xavier Mauvois, Patrick Martin, Salima Aroua, Karine Rousseau, Sylvie Dufour

**Affiliations:** ^1^ Muséum National d’Histoire Naturelle, Unité Mixte de Recherche Biologie des Organismes et Ecosystèmes Aquatiques (UMR BOREA), Biology of Aquatic Organisms and Ecosystems, Centre National de la Recherche Scientifique (CNRS), Institut de Recherche pour le Développement (IRD), Sorbonne Université, Paris, France; ^2^ Université Le Havre Normandie - Stress Environnementaux et Biosurveillance des milieux aquatiques UMR-I 02SEBIO -FR CNRS 3730 SCALE, Le Havre, France; ^3^ Conservatoire National du Saumon Sauvage (CNSS), Chanteuges, France

**Keywords:** corticotropin-releasing hormone, phylogeny, synteny, tissue distribution, vertebrates, eel, salmon

## Abstract

Corticotropin-releasing hormone (CRH) was discovered for its role as a brain neurohormone controlling the corticotropic axis in vertebrates. An additional *crh* gene, *crh2*, paralog of *crh* (*crh1*), and likely resulting from the second round (2R) of vertebrate whole genome duplication (WGD), was identified in a holocephalan chondrichthyan, in basal mammals, various sauropsids and a non-teleost actinopterygian holostean. It was suggested that *crh2* has been recurrently lost in some vertebrate groups including teleosts. We further investigated the fate of *crh1* and *crh2* in vertebrates with a special focus on teleosts. Phylogenetic and synteny analyses showed the presence of duplicated *crh1* paralogs, *crh1a* and *crh1b*, in most teleosts, resulting from the teleost-specific WGD (3R). *Crh1b* is conserved in all teleosts studied, while *crh1a* has been lost independently in some species. Additional *crh1* paralogs are present in carps and salmonids, resulting from specific WGD in these lineages. We identified *crh2* gene in additional vertebrate groups such as chondrichthyan elasmobranchs, sarcopterygians including dipnoans and amphibians, and basal actinoperygians, Polypteridae and Chondrostei. We also revealed the presence of *crh2* in teleosts, including elopomorphs, osteoglossomorphs, clupeiforms, and ostariophysians, while it would have been lost in Euteleostei along with some other groups. To get some insights on the functional evolution of the *crh* paralogs, we compared their primary and 3D structure, and by qPCR their tissue distribution, in two representative species, the European eel, which possesses three *crh* paralogs (*crh1a*, *crh1b*, *crh2*), and the Atlantic salmon, which possesses four *crh* paralogs of the crh1-type. All peptides conserved the structural characteristics of human CRH. Eel *crh1b* and both salmon *crh1b* genes were mainly expressed in the brain, supporting the major role of *crh1b* paralogs in controlling the corticotropic axis in teleosts. In contrast, *crh1a* paralogs were mainly expressed in peripheral tissues such as muscle and heart, in eel and salmon, reflecting a striking subfunctionalization between *crh1a* and *b* paralogs. Eel *crh2* was weakly expressed in the brain and peripheral tissues. These results revisit the repertoire of *crh* in teleosts and highlight functional divergences that may have contributed to the differential conservation of various *crh* paralogs in teleosts.

## Introduction

Corticotropin-releasing hormone (CRH), first named CRF (F for factor) until its characterization, is the hypothalamic neurohormone of the corticotropic axis involved in the control of stress response. CRH control the production by the pituitary of corticotropin (ACTH), which itself controls the production of corticosteroids by the adrenals in mammals, birds and reptiles, and by the interrenals in amphibians and teleosts. Being able to activate other neuroendocrine axes, such as the thyrotropic and somatotropic axes, under environmental and internal stimuli, CRH has been proposed as the central coordinator of endocrine activation during life transitions such as metamorphosis in amphibians and teleosts, and egg hatching in birds and reptiles [for reviews: (1, 2)].

Initial evidence of the existence of CRH was reported in 1955 (3, 4), and over 25 years were needed to isolate CRH peptide in sheep (5). Subsequently, the ovine CRH precursor, named also prepro-CRH, was cloned and its primary structure described (6). Soon after, the characterization of CRH in rat (7, 8) and human (9) revealed the strong structural and molecular conservation of the CRH peptide that consists of an alpha helical peptide of 41 amino-acids (aa) which is C-terminally amidated and that is produced from the cleavage of a larger precursor ranging from 160 to 210 aa depending on the species [for review (10)]. In mammals, CRH-expressing neurons were found in paraventricular nucleus (PVN) with the majority of neurons projecting to the median eminence (11).

Other peptides showing similarities with CRH were characterized: sauvagine from the skin of neotropical frog *Phyllomedusa sauvagei* (12), urotensin-I from the urophysis of the white sucker *Catostomus commersoni* (13) and urocortin from rat midbrain (14), all now considered as “urocortin”, encoded by *ucn* (named *ucn1*, 15), a paralogous gene of *crh* [for review (16)]. Two other CRH-related peptides, named urocortin 2 (17) and urocortin 3 (18) were later found thanks to the completion of the human genome project. According to current evolutionary scenarios, *ucn1* would be in fact more closely related to *crh* than to *ucn2* and *ucn3* [for review (16); (15, 19)]. Only one *crh* gene (*crh*) had been identified in vertebrates, until the discovery in 2011 of a second *crh* in the elephant shark *Callorhinchus milii* (20). This second *crh* gene (named *crh2*) was then identified in most vertebrate groups (21), a result confirmed by other studies (15, 19). Phylogenetic and syntenic analyses led to the hypothesis that the paralogs, the “classical” *crh* (also named *crh1* for clarification, 15) and *crh2*, as well as *ucn1* likely arose during the two rounds (1R/2R) of whole genome duplication (WGD) that occurred early in the vertebrate lineage (15, 21). In addition, it had been proposed that *crh2* would have been lost repeatedly during vertebrate radiation, in teleosts, in amphibians and in placental mammals (15, 21, 22). Duplicated *crh (crh1a* and *crh1b)* were found in teleosts likely resulting from the teleost-specific WGD (3R) (15, 22). A further duplication of *crh1a* and *crh1b* resulting from salmonid-specific WGD (4R) led to four *crh1* paralogs in salmonids (22, 23). Conservation of paralogs after duplication may be related to evolutionary selection for either amplification of initial function, sharing of initial pleiotropic functions (subfunctionalization) or emergence of a new function (neofunctionalization) [for review (24)].

In teleosts, as in other vertebrates, CRH conserved its role as a major stimulator of pituitary ACTH release in response to stress [for reviews (25, 26)]. CRH is also involved in metabolism, food intake [for review (27)], immunity [for review (28)] and locomotor activity [for review (29)]. In teleosts, CRH not only controls the corticotropic axis but is also a potent activator of the thyrotropic axis as in amphibians and birds. This suggests that CRH is able to control several physiological functions in teleost fish such as stress, osmoregulation, metabolism and may play an important role as central coordinator of the activation of endocrine axes for developmental transitions and adaptation to environmental changes [for reviews (1, 2)].

In the present study, we investigated *crh* paralogs in vertebrates and specially the fate of *crh2* which was previously assumed to be lost in teleosts. In addition, to get some insights into the functional evolution of the *crh* paralogs, we analyzed their sequences, 3D structures and tissue distributions in two representative teleosts: a basal teleost, the European eel (Elopomorph), in which we revealed the presence of three *crh* paralogs (*crh1a, crh1b* and *crh2*), and the Atlantic salmon (Salmoniformes) which possesses four *crh* paralogs, all of the *crh1*-type. Our data show that teleost *crh* paralogs evolved distinct expression patterns and likely diverse functions.

## Materials and Methods

### *In silico* Identification of Corticotropin-Releasing Hormone Genes

Corticotropin-releasing hormone (*crh*) genes were sought in representative vertebrates with a special focus in the actinopterygian lineage. Sequences from the most closely related paralog to *crh*, *ucn1*, were also included. We screened the genomes of 70 vertebrate species. Gene sequences were retrieved from genome assemblies, either using GenBank gene prediction, or by an exhaustive Blast search against GenBank and UCSC genomic databases, and GenBank, UCSC and PhyloFish transcriptomic databases (30). We used genome assemblies and RNA libraries to search for non-annotated *crh-like* genes or to confirm gene loss. Coding sequence (CDS) of the prepropeptide were manually annotated by comparison with orthologous genes using CLC Main Workbench (QIAGEN). Sequences references and annotations are provided in **Table S1**. Phylogenetic analysis, supported by synteny analysis, allowed us to identify orthologs/paralogs and name or rename *crh* genes, accordingly. For gene nomenclature of WGD paralogs in teleosts, we used in the present study the letters “a/b” for teleost-specific 3R-duplicated genes, according to Zfin nomenclature conventions, and the symbols “α/β” for salmonid-specific 4R-duplicated genes according to Robertson and colleagues (31).

The signal peptide was predicted using SignalP 4.0 browser (32) and peptide cleavage site using both Modpred and NeuroPred webbrowser (33, 34).

### Phylogenetic Analysis

Phylogenetic analyses were performed using 166 CRH and UCN1 sequences from 48 vertebrates. CRH/UCN1 mature peptides are highly conserved peptides, which makes it difficult to extract information from their amino-acid sequences, so we used the prepropeptide amino-acid sequences to infer the phylogenetic tree. Multiple sequence alignments of the CRH/UCN1 protein family were performed using the slow algorithm available on CLC workbench and further manually edited based on conserved amino-acid sequences.

The tree topology was inferred with a maximum likelihood analysis using PhyML 3.0 on the web browser of ATGC Montpellier bioinformatics platform and Seaview (35–37). The WAG substitution matrix (38) was chosen to infer the trees of CRH family. Strength of branch nodes was evaluated by both aLRT and bootstrap using 100 replicates.

### Synteny Analysis

To further resolve *crh* gene evolution in teleosts, the synteny of *crh* and *ucn1* genes was investigated in actinopterygians, using a basal non-teleost actinopterygian, a Polyperidae, the reedfish (*Erpetoichthys calabaricus*) as template. The four chromosomes corresponding to the *crh1/crh2/ucn1* tetraparalogon were identified in reedfish by comparing with the *crh1/crh2/ucn1* paralogous chromosomes of spotted gar (15). Conserved neighboring gene families of *crh1/crh2/ucn1* were identified by comparing manually NCBI predicted genes lists on the *crh1/crh2/ucn1* chromosomes of reedfish (**Figure S1**). The following teleost species were studied: representatives of two basal groups, an elopomorph, European eel (*Anguilla anguilla*) and an osteoglossomorph, arowana (*Scleropages formosus*); a Clupeidae, herring (*Denticeps clupeoides*), a Cyprinidae, zebrafish (*Danio rerio*), an Esocidae, pike (*Esox lucius*) and a Salmonidae, Atlantic salmon (*Salmo salar*). Neighboring gene families with members inherited from 1 and 2R vertebrate WGD, 3R teleost WGD and 4R salmonid WGD were selected to illustrate the *crh*-gene paralogon evolution. Additional genes were used to further discriminate 3R- and 4R-paralogons. Neighboring gene identity was confirmed by examining close gene neighborhood using the genome browser, Genomicus (39). Non-predicted genes were sought by extensive blast against genome assemblies to confirm the gene loss. References of the gene neighborhood of the *crh/ucn1* family is provided in **Table S2**. The neighboring genes of *crh2* in arowana were reexamined together with those of another osteoglossomorph, *Paramormyrops kingsleyae* and are given in **Table S2**.

### 3D-Structure Prediction

The structure of human, eel and salmon CRH peptides was predicted using iTASSER browser (40, 41). Protein model quality was assessed using MoldFold (42). All the five resulting models predicted by iTASSER, showed high quality score. For each peptide, the top model ranked was rendered using ChimeraX v1.3 (43). Logo sequences of the CRH precursors were generated from the alignment generated for the phylogenetic analysis using CLC Main Workbench (QIAGEN).

### Fish and Tissue Samples

Female European eels (*A. anguilla*) were at the prepubertal “silver” stage, corresponding to the end of the continental stage of the eel life cycle, previous to migration to the ocean for reproduction. They were purchased from Gebr. Dil import-export BV (Akersloot, The Netherlands) and transferred to MNHN, France. Animals were anesthetized by cold and then killed by decapitation under the supervision of authorized person (KR; No. R-75UPMC-F1-08) according to the protocol approved by Cuvier Ethic Committee France (No. 68–027). The tissues were dissected, incubated in RNA later overnight at 4°C and stored at -20°C until RNA extraction.

For Atlantic salmon (*S. salar*), we reused the tissue samples collected for a previous study (44). These tissues had been collected from mixed sex (5 males and 5 females) juvenile fish of the Loire-Allier population raised indoor under natural water, temperature, and photoperiod conditions, at the Conservatoire National du Saumon Sauvage (CNSS) (44).

The following tissues, dissected according to (44, 45), were analyzed in both species: whole brain, pituitary, gill, heart, liver, spleen, kidney, intestine, fat, muscle, skin and gonad. For detailed brain distribution the following tissues were also analyzed: olfactory bulbs, telencephalon, mesencephalon, diencephalon, optic tectum, cerebellum, medulla oblongata as well as saccus vasculosus, epiphysis, pituitary and retina.

### Quantitative Gene Expression Analysis

#### RNA Extraction and cDNA Synthesis

Total tissue RNA was extracted using Trizol according to the manufacturer recommendations. Tissues were homogenized in Trizol with steel beads, twice at 30Hz for 2-5 min, using a TissueLyzer II (Qiagen). Total RNA concentration was measured using a nanodrop spectrophotometer (Thermo Fisher Scientific) and treated with DNase I (Roche) according to the manufacturer’s instructions at 37°C for 20 min. DNase I was inactivated and removed by phenol extraction.

Complementary DNA was generated from 750 ng of denatured total RNA (at 65°C for 5 min) and 75 ng random primers using the superscript III (Invitrogen) under the following conditions: a primer hybridization step at 25°C for 10 min, followed by an extension step at 50°C for 60 min and an inactivation step at 70°C for 15 min. Two no-reverse transcriptase controls for potential DNA contamination were performed using total RNA from either brain or muscle in the same reaction conditions but without superscript III: no product or non-specific products were amplified by qPCR.

#### Quantitative Real-Time PCR

Many primer sets for each *crh* gene of eel and salmon were designed using primer 3 browser (**Table S3**). The design of primer sets for each gene were confined to the sequence parts showing nucleotide divergence between paralogs, which was especially critical for the pairs of salmon *crh1 4R-*paralogs that showed strong identity between each other. Each primer set was first tested on pools of tissues expressing the *crh* paralogs (brain and muscle). These tissues were used to produce qPCR standards: the standards were serial dilutions of cDNA from pooled samples of eel brain for eel *crh1b* and *crh2*, of eel muscle for eel *crh1a*, of salmon brain for salmon *crh1bα* and *crh1bβ* and of salmon muscle for salmon *crh1aα* and *crh1aβ*. Standard dilutions were run in duplicate to draw a calibration curve and measure amplification efficiency of the different primer sets. Primer sets were chosen according to the two following conditions: 1) they showed a good amplification efficiency (**Table S3**), 2) gene amplification efficiency was confirmed with two different primer sets positioned at different gene regions (same cycle quantification (Cq) for a same standard dilution) and 3) the Tm of the amplicon could be discriminated from the one resulting from primer-dimer amplification.

Quantitative PCR assays were performed using the LightCycler (Roche) with the LightCycler FastStart Master plus SYBR Green I kit (Roche) as recommended by the manufacturer and 500 nM of each primer. Each sample was run in duplicate. The PCR conditions were 95°C for 10 min followed by 50 cycles at 95°C for 5 sec, 60°C for 10 sec and 72°C for 5 sec. The specificity of amplified qPCR products was checked by performing melting curve analyses and by sequencing the amplicon. Transcript quantity was calculated using the LightCycler software from quantification cycle (Cq) determined by the automatic second derivative method, according to the calibration curve method. Samples were considered at the limit of detection when amplification was obtained after 42 Cq and a minimal value was assigned which was the lowest detectable dose of respective standard. Data are expressed as arbitrary units of gene transcript level/total RNA level. Results (means ± SEM) are presented as percentage per tissue for the tissue comparison, according to (46, 47).

## Results

### Identification of *crh/ucn1* Genes in Representative Vertebrates and Phylogenetic Analysis

**Figure 1:**
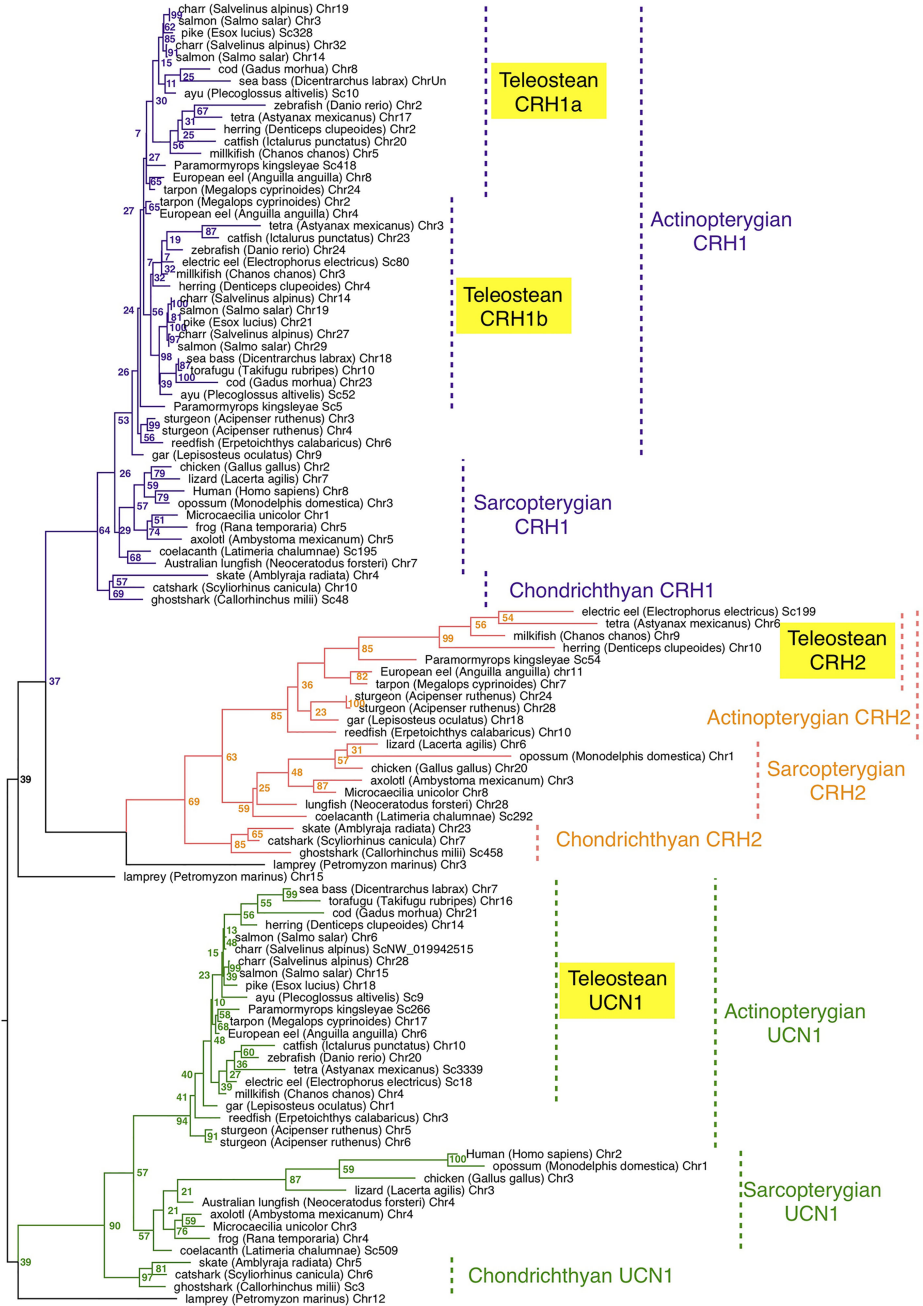
Maximum-likelihood phylogenetic tree of CRH prepropeptide amino acid sequences of vertebrate representatives. Phylogenetic relationships of CRH was inferred using the PhyML algorithm with the WAG substitution matrix and the best nearest neighbour interchange (NNI) and Subtree Pruning and Regrafting (SPR) improvement algorithm. Numbers at the node indicate the confidence percent of 100 bootstrap replications. The three gnathostome monophyletic clades are indicated with different branch colors and the corresponding CRH clade names are indicated using the same colour. Taxonomic group names are indicated at the right of the tree. The list of corresponding gene references is provided in **Table S1**.

The presence of *crh/ucn1* genes was investigated in 70 vertebrates including 7 chondrichthyans, 14 sarcopterygians and 48 actinopterygians, and 252 sequences were retrieved from the genome assemblies of representative vertebrates (**Table S1**). A phylogenetic tree was inferred by maximum likelihood analysis from prepro-CRH/UCN1 amino-acid sequences (**Figure 1**); a second phylogenetic analysis was performed with a special focus on a larger number of actinopterygian species (**Figure S2**). Vertebrate CRH/UCN1 sequences clustered into three groups, including sequences of CRH1, CRH2 and UCN1 respectively (**Figure 1**). Three genes were present in a basal vertebrate (a cyclostome, the lamprey, *Petromyzon marinus*) as previously reported (19) and their amino-acid sequences branched at the basal position of each of the gnathostome CRH1, CRH2 and UCN1 clusters in agreement with vertebrate phylogeny (**Figure 1**).

In chondrichthyans, three genes, *crh1, crh2* and *ucn1*, were present in a holocephalan (elephant shark, *C. milii*) as previously described, and were also retrieved in the present study in representatives of elasmobranchs, including selachians such as catshark, *Scyliorhinus canicula* and Batoidae such as the thorny skate, *Amblyraja radiata* (**Table S1**). Chondrichthyan amino-acid sequences branched at the basis of the osteichthyan CRH1, CRH2 and UCN1 clusters, respectively, also in accordance with vertebrate phylogeny (**Figure 1**).

In basal sarcopterygians, we found three genes (*crh1, crh2* and *ucn1*) in actinistians (coelacanth, *Latimeria chalumae*) as previously reported, branching at the basis of the sarcopterygian sequences. We predicted two to three genes in the current status of the lungfish (Dipnoi) genome assembly (**Table S1**). The genome of the Australian lungfish (*Neoceratodus forsteri*) encoded indeed two full-length genes, corresponding to *crh2* and *ucn1* according to our phylogenetic analysis (**Figure 1**), as well as one *crh1* pseudogene showing a frameshift due to indels at two different positions on the CDS. In the West African lungfish (*Protopterus annectens)* only two genes (*crh1* and *ucn1*) could be retrieved (**Figure S2**; **Table S1**). In amphibians, we retrieved only two genes (*crh1* and *ucn1*) in various anuran species (such as *Rana temporaria*) as previously reported for Xenopus. In contrast we identified a third gene, corresponding to *crh2* according to our phylogenetic analysis, in representative species of other amphibian groups, Gymnophiona (*Microceacilla unicolor*), and Caudata (axolotl, *Ambystoma mexicanum*) (**Figure 1**). The three genes (c*rh1, crh2* and *ucn1*) were present in representative species of the various groups of sauropsids including birds, as recently reported (48), as well as in protherian and metatherian mammals, in agreement with previous studies (21) (**Figure 1**; **Table S1**).

In basal actinopterygians, we retrieved three genes, corresponding to *crh1*, *crh2* and *ucn1*, in a representative of cladistian Polypteridae (reedfish, *Erpetoichthys calabricus*), as well as in two representative species of another non-teleost group, holosteans (spotted gar, *Lepisosteus oculatus*, as previously reported; and bowfin, *Amia calva*) (**Figures 1**, **S2**). In chondrostean species, including Polyodontinae (paddlefish, *Polyodon spathula*), and Acipenserinae (sterlet sturgeon, *Acipenser ruthenus*), we found six genes corresponding to duplicated genes for *crh1*, *crh2* and *ucn1* (**Figures 1**, **S2**) likely resulting from the polyploidization of the genome in these species.

In basal teleosts, four genes were predicted in elopomorphs, Anguillidae (European, American and Japanese eels, *Anguilla sp*), and Megalopydae (tarpon, *Megalops cyprinoides*) (**Table S1**), corresponding to duplicated *crh1* (*crh1a* and *crh1b*), a single *crh2* and a single *ucn1*, according to our phylogenetic analyses (**Figures 1**; **S2**). *Crh1b* paralog was present in all teleost species investigated in the present study, while *crh1a* could not be found in the fugu (*Takifugu rubripes*) in agreement with previous studies (22). Among clupeiforms, full-length *crh1a* was found in Denticipitidae (dendicle herring), and in Clupeidae (Atlantic herring, *Clupea harengus* and American shad, *Alosa sapidissima*), while a *crh1a* pseudogene was retrieved in an Engraulidae (anchovy, *Coilia nasus*) (**Figure S2**; **Table S1**). No *crh1a* sequence could be found in another Clupeidae (pilchard, *Sardina pilchardus*) nor in a gymnotiform (electric eel, *Electrophorus electricus*), but this may be due to the current status of their genome assemblies. We identified *crh2*, previously assumed to be lost in teleosts, as a single gene not only in elopomorphs as mentioned above but also in osteoglossomorphs, including Mormyridae (elephant fish, *P. kingsleyae)*, Gymnarchidae (aba, *Gymnarchus niloticus*), and Osteoglossidae (arapaima, *Arapaima gigas*), and partial sequence in arowana, as well as in Clupeiformes (denticle and Atlantic herrings, American shad; Alice shad, (*Alosa alosa)*, pilchard, anchovy) and in several ostariophysian groups including Gonorynchiformes (milkfish *Chanos chanos*), Characiformes (Mexican tetra, *Astyanax mexicanus*) and Gymnotiformes (electric eel) (**Figure 1** and **S2**; **Table S1**). In contrast, we could not retrieve any *crh2* gene in some other ostariophysian groups, Silurifomes and Cypriniformes. *Crh2* gene could not be found either in any euteleostean species investigated, including esociforms, salmoniforms, galaxiiforms, osmeriforms, gadiforms, nor in any of the acanthopterygian groups. A single *ucn1* gene could be retrieved in all teleost genomes investigated, and was duplicated in salmonids and some polyploid cyprinids, in relation to the further genome duplication in these lineages (**Figures 1** and **S2**; **Table S1**). We also retrieved two *ucn1* genes in denticle herring, one on the chromosome 14 and the other one on an unplaced scaffold (**Tables S1**, **S3**). These two genes showed 99.98% nucleotide identity indicating they might result either from recent sequential duplication or from a genome assembly artefact. In the present study, only the UCN1 carried by the chromosome 14 was further considered.

### Synteny Analysis of *crh* Family Genes in Actinopterygians

**Figure 2:**
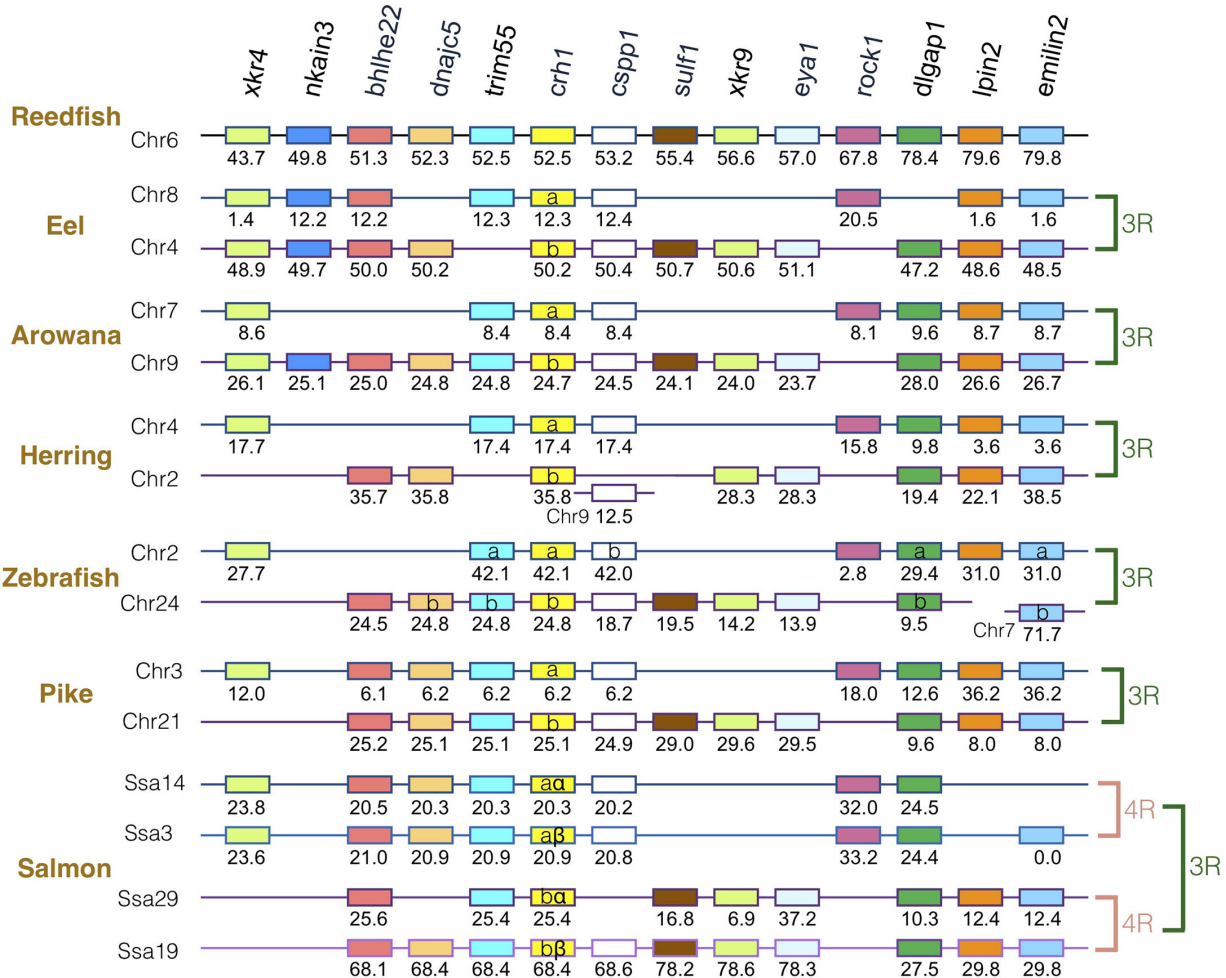
Synteny of crh1 genomic region in actinopterygians. Crh1 gene neighborhood is compared between teleost representatives, eel, arowana, herring, zebrafish, pike and salmon. The polypteriform actinopterygian, reedfish, was used as model of the gene arrangement prior the teleost genome duplication 3R. Chromosomes are indicated by Chr or Ssa for salmon. Gene position are indicated under the genes. Loss of *crh1* gene is indicated with a red cross. Neighboring genes non-represented are considered as lost. Gene references are presented in **Table S3**. Letters a and b indicate the 3R-teleost duplicated paralogs and the symbols α and β the 4R-salmonid duplicated paralogs. Members of gene families conserved on the vertebrate tetraparalogon (issued from 1R/2R WGD) are with the same color in **Figures 3**–**4**.

**Figure 3:**
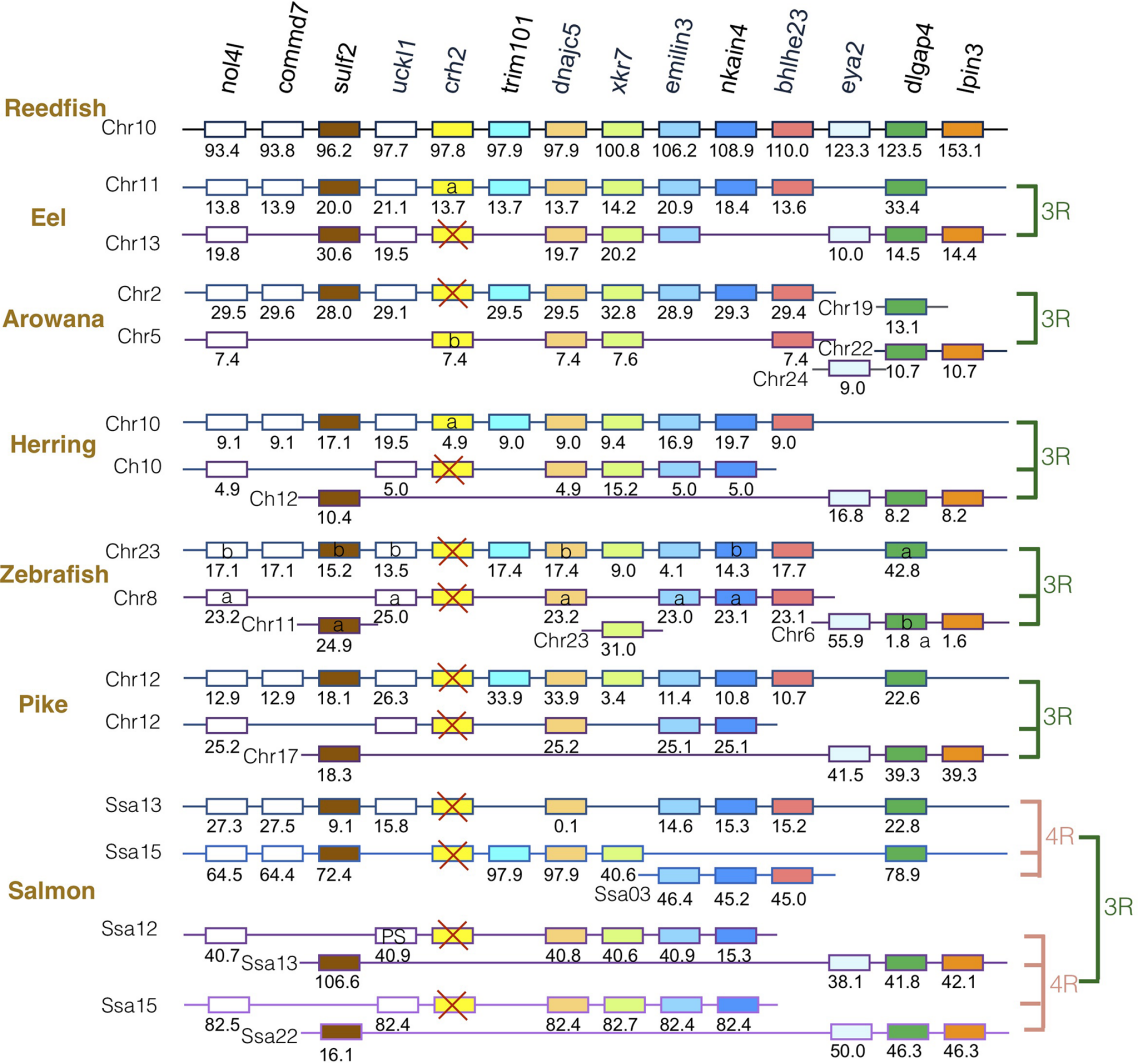
Synteny of crh2 genomic region in actinopterygians. Crh2 gene neighborhood is compared between teleost representatives, eel, arowana, herring, zebrafish, pike and salmon. The polypteriform actinopterygian, reedfish, was used as model of the gene arrangement prior the teleost genome duplication 3R. Chromosomes are indicated by Chr or Ssa for salmon. Gene position are indicated under the genes. Loss of *crh2* gene is indicated with a red cross. Neighboring genes non-represented are considered as lost. Gene references are presented in **Table S3**. Letters a and b indicate the 3R-teleost duplicated paralogs and the symbols α and β the 4R-salmonid duplicated paralogs. Members of gene families conserved on the vertebrate tetraparalogon (issued from 1R/2R WGD) are with the same color in **Figures 3**, **4**.

**Figure 4:**
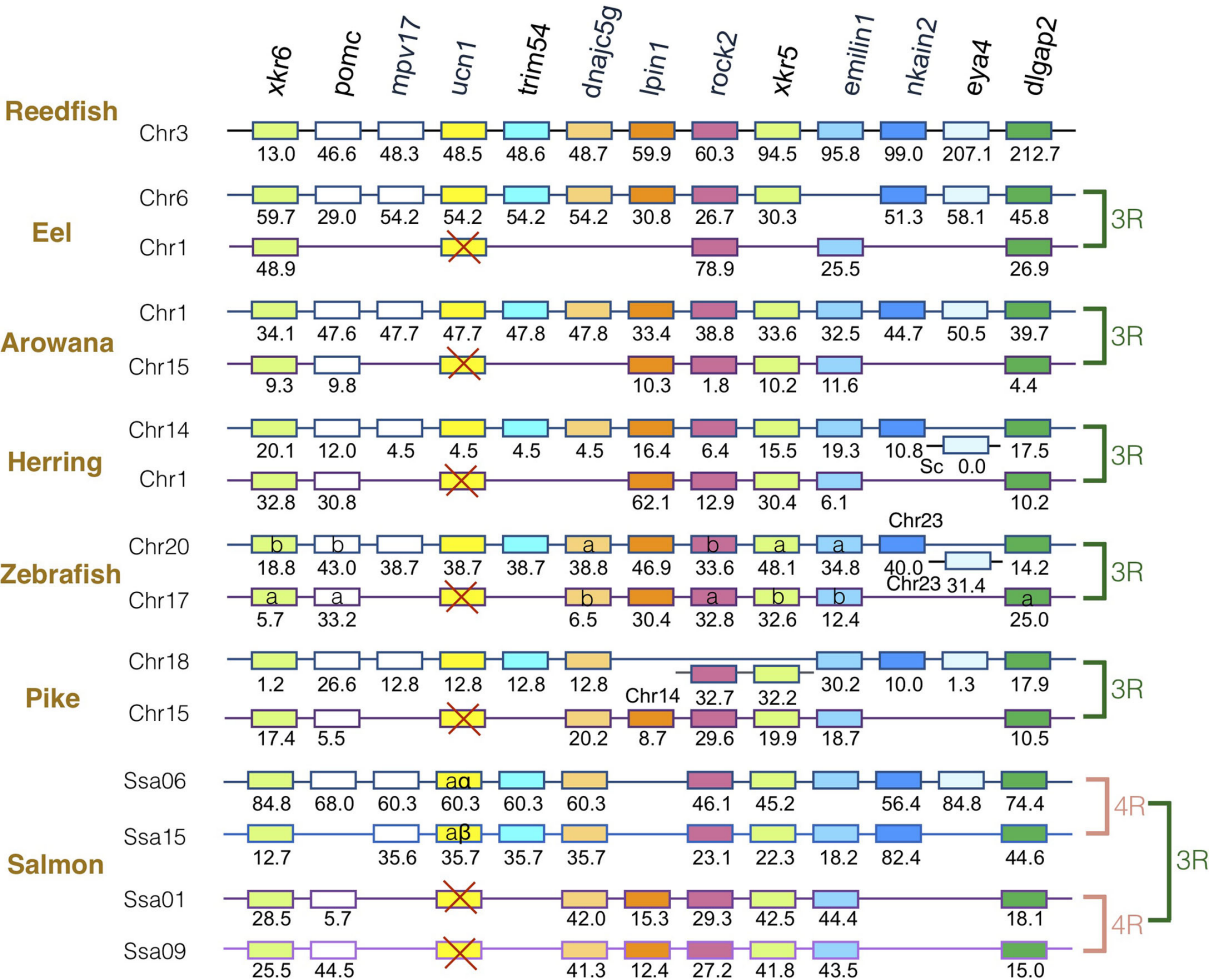
Synteny of ucn1 genomic region in actinopterygians. Ucn1 gene neighborhood is compared between teleost representatives, eel, arowana, herring, zebrafish, pike and salmon. The polypteriform actinopterygian, reedfish, was used as model of the gene arrangement prior the teleost genome duplication 3R. Chromosomes are indicated by Chr or Ssa for salmon. Gene position are indicated under the genes. Loss of *ucn1* gene is indicated with a red cross. Neighboring genes non-represented are considered as lost. Gene references are presented in **Table S3**. Letters a and b indicate the 3R-teleost duplicated paralogs and the symbols α and β the 4R-salmonid duplicated paralogs. Members of gene families conserved on the vertebrate tetraparalogon (issued from 1R/2R WGD) are with the same color in **Figures 2**–**3**.

The gene family members *xkr4/5/6/7/9*, *nkain2/3/4*, *bhlhe22/23*, *dnajc5/dnajc5g*, *trim54/55/101*, *sulf1/2*, *eya1/2/4*, *rock1/2*, *dlgap1/2/4*, *lpin1/2/3*, and *emilin1/2/3* were found to surround the *crh1*/*crh2/ucn1* family genes on the tetraparalogous chromosomic regions of the reedfish (**Figures 2**–**4** and **S1**), in agreement with their 1R and 2R origin. Additional neighboring gene families were used to further analyze the impact of 3R and 4R in teleosts, such as *cspp1* on *crh1* paralogon, *nol4l*, *commd7* and *uckl1* on *crh2* paralogon, *pomc* and *mpv7* on *ucn1* paralogon. The synteny comparison among actinopterygians showed that the *crh1, crh2* and *ucn1* paralogons were duplicated by the teleost 3R WGD and further duplicated by salmonid 4R WGD (SS4R) (**Figures 2**–**4**).

Concerning *crh1* paralogon (**Figure 2**), 3R-duplicated *crh1* paralogs (*crh1a* and *crh1b*) were conserved in eel, arowana, zebrafish, pike, as well as 3R-duplicated neighboring gene paralogs such as *cspp1* and *dlgpa1*. The salmonid 4R WGD led to quadruplicated *crh1* paralogs (*crh1aα, crh1aβ*, *crh1bα, crh1bβ*) as well as quadruplicated neighboring genes *bhlhe22, trim55* and *dlgap1*.

Concerning *crh2* paralogon (**Figure 3**), some translocation events occurred in this genomic region. It could still be inferred that some neighboring genes, such as *nol4l, sulf2, dnajc5, nkain4* and *dlgap4*, were conserved as duplicates after 3R and as quadruplicate after 4R. In contrast, a single *crh2* gene was present in eel, arowana and herring, and we could not find any *crh2* gene in the genome assemblies of zebrafish, pike and salmon. Comparison of neighboring genes conserved as single paralogs allowed to infer that a different *crh2* paralog was conserved in eel and herring *versus* in arowana. The *crh2* paralog named *crh2a* conserved in eel and herring was located in the paralogon bearing the single conserved paralog of *commd7*, *trim101*, *nkain4*, while the *crh2* paralog named *crh2b* conserved in arowana was located on the other paralogon. Since the *crh2* gene retrieved from the arowana genome assembly is fragmented, we also examined the *crh2* gene neighborhood in another osteoglossomorph species belonging to the Mormyridae, the *P. kingsleyae* for which *crh2* gene encodes a putative functional Crh2 (**Table S2**). This confirmed the presence of a *crh2* (*crh2b*) gene in the other 3R- ohnologous region than the one carrying eel and herring *crh2* (*crh2a*) gene. The lack of *crh2* in pike and salmon suggests that a loss of *crh2* already occurred in their common ancestor and therefore no impact of 4R. We observed a chromosome fusion between the two *crh2* genomic regions in herring, pike and salmon that may have occurred prior the radiation of Clupeocephala. Such genome reorganization between paralogous regions have already been described for other gene duplicated paralogs such as the melatonin receptors (47). These events were related to intense rate of interchromosomal rearrangements that occurred in some lineages during the rediploidization process after the 3R (49).

Concerning *ucn1* paralagon, some neighboring genes such as *xkr6, rock2, emilin1, dlgap2* were conserved as duplicates in eel, arowana, herring, zebrafish, and pike and as quadruplicates in salmon, reflecting the impact of 3R and 4R on this genomic region. In contrast, *ucn1* was found as a single gene in eel, arowana, herring, zebrafish and pike. This single *ucn1* was located in all these species on the paralogon bearing the single conserved paralog of *mpv7, trim54, nkain2 and eye4* neighboring genes, indicating that the same *ucn1* paralog was conserved in the different teleost species. Synteny analysis indicated that this single *ucn1* gene was inherited by salmonids and duplicated by the 4R.

### Comparison of Eel and Salmon CRH Prepropeptides and Peptides Sequences

Both European eel *crh1a* and *crh1b* paralogous genes, located on the chromosome 8 and 4 respectively, code for 164 amino acid (aa) precursors, prepro-Crh1a and prepro-Crh1b respectively, including a 24 aa signal peptide required for the hormone release, a 97 aa cryptic region with at its C-terminus a proteolytic cleavage site at the position R^121^, and the 41 aa C-terminal region coding for CRH peptide with a C-terminal Gly-Lys amidation site. At the difference of the prepro-Crh1b, the prepro-Crh1a showed putative additional cleavage sites at the positions R83 and R103. Japanese eel *crh* (BAP90537), previously characterized (50), is the ortholog to European eel *crh1b*. The eel *crh2* gene located on the chromosome 11 encodes a 149 aa prepropeptide including a 23 aa signal peptide, a cryptic region of 81 aa at the N-terminus and a proteolytic cleavage site at the position R^104^, and the C-terminal region encoding a 43 aa mature Crh2 with a C-terminal amidation site. The prepro-Crh2 shows a putative additional cleavage site at the position R^33^. The eel *ucn1* gene located on chromosome 6 codes for a 168 aa prepropeptide, including a 24 aa signal peptide, a 101 aa cryptic region, the C-terminal region coding for a 41 aa Ucn1 peptide with a C-terminal amidation site.

**Figure 5:**
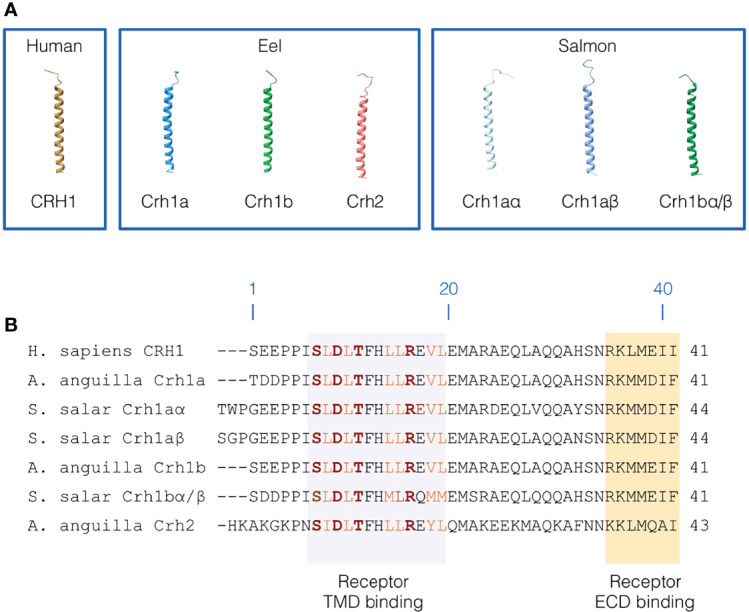
Primary and 3D structure of CRH peptides. **(A)** 3D structure of human CRH1 peptide, and the three CRH peptides (3R-Crh1a and b, Crh2) in European eel and four CRH peptides (4R-Crh1aα and aβ, 4R-Crh1bα/bβ which have the same sequence) in Atlantic salmon. CRH peptide structures were modelled using iTASSER. **(B)** Sequence alignment of human CRH1, eel 3R-Crh1a and b and Crh2, and Atlantic salmon 4R-Crh1aα, aβ, and 4R-Crh1bα/bβ. Residue numbering are based on human CRH1. The region of CRH peptides interacting with the CRH receptor transmembrane domain are highlighted in light purple and the region of CRH peptides interacting with the CRH receptor extracellular domain are highlighted in orange. Conserved hydrophobic residues involved in the receptor binding are indicated in orange and the residues involved in electrostatic interaction with the receptor are indicated in red and bold.

The two Atlantic salmon *crh1aα* and *crh1aβ* 4R-paralogous genes (located on the chromosomes 14 and 3, respectively) encode 171 and 170 aa prepropeptides, including a 24 aa signal peptide, a 100 and 101 aa cryptic region, respectively, and a C-terminal sequence encoding a 44 aa Crh1 mature peptide with a C-terminal amidation site. The two salmon *crh1bα* and *crh1bβ* 4R-paralogous genes (located on chromosomes 29 and 19) both encode for a 167 aa prepropeptide, including a 24 aa signal peptide, and a C-terminal sequence encoding for a 41 aa Crh1 mature peptide with a C-terminal amidation site. The two salmon *ucn1α* and *ucn1β* 4R-paralogous genes (located on chromosomes 6 and 15) code for 165 and 162 aa prepropeptides respectively, including a 24 aa signal peptide, a cryptic region of 98 aa and 95 aa respectively, and a 41 aa Ucn1 peptide with a C-terminal amidation site (**Figures 5** and **S3**).

Comparison with salmonid cDNA sequences from previous studies indicates that: - Atlantic salmon “*crh”* (DY733166) (22, 51, 52), Atlantic salmon *“crf1b1”* (23), Atlantic salmon “*crf-b1*” (53), and trout, *Oncorhynchus mykiss*, *crh* (NM_001124286) (46) correspond to salmon *crh1bα* in our study; - Atlantic salmon “*crf1b2”* (23), Atlantic salmon “*crf-b2*” (53), and trout “*crh2”* (AY156929 and NM_001124627) (46, 54) correspond to salmon *crh1bβ*.

The aa sequences of European eel 3R-paralogs Crh1a and Crh1b shared 65 and 90% identity for the prepropeptides and the peptides, respectively. Eel prepro-Crh1a and prepro-Crh1b shared 38 and 42% identity with human prepro-CRH1, and 19 and 16% identity with human prepro-UCN1. Eel Crh1a and Crh1b peptides shared 86 and 95% identity with human CRH peptide, and 49 and 44% identity with human UCN1 peptide. When comparing eel Crh1a and b with eel Crh2 the percentage of identity fell down to 18 and 19% for the prepropeptides and 49% for the peptides. Eel Crh2 peptide shared higher identity with other Crh2 peptides: 88% for the tarpon, 39-67% for the other teleosts, 72-81% for the non-teleost actinopterygians, 41-67% for the sarcopterygians, 56-60% for the chondrichthyans. Eel prepro-Crh2 shared 15 and 11% identity with human prepro-CRH1 and prepro-UCN1, respectively, and eel Crh2 peptide 54 and 36% identity with the human CRH1 and UCN1 peptides. Eel Ucn1 shows 50 and 21% identity with human prepro-UCN1 and prepro-CRH1, respectively, and 60 and 56% identity for the corresponding peptides.

Atlantic salmon 4R-Crh1aα and Crh1aβ paralogs shared 86 and 89% identity between the prepropeptides and the peptides, respectively and the 4R-Crh1bα and Crh1bβ paralogs showed 93 and 100% identity between the prepropeptides and the peptides, respectively. When Crh1aα/β and Crh1bα/β pairs of paralogs were compared, the identity fell down to the range of 56 to 58% between prepropeptides, and to the range of 66 to 68% between peptides. The salmon Crh1aα and Crh1aβ prepropeptides showed 36 and 35% identity with the human prepro-CRH1, respectively, and the Crh1aα and Crh1aβ peptides shared 77 and 82% identity with human CRH1 peptide. The salmon Crh1bα and Crh1bβ prepropeptide showed 38 and 37% identity with the human prepro-CRH1 respectively and Crh1bα/bβ peptides showed 76% identity with human CRH1 peptide, respectively. The percentage of identity fell down when compared with human prepro-UCN1 (18-19%) and UCN1 peptide (40-43%). The two salmon 4R-Ucn1 showed higher identity with human UCN1 (21 and 22% for the prepro-UCN1 respectively, and both 64% for UCN1 peptide) than with human CRH1 (18 and 17% for the prepropeptides, respectively, and both 56% for the peptides).

Using a sequence logo, the overall comparison of CRH1, CRH2 and UCN1 prepropeptides sequences from various vertebrates used in the phylogeny (**Figure 1**; **Table S1**), revealed a high sequence conservation of CRH1, CRH2 and UCN1 peptides and of the signal peptides. The conservation of some parts of the cryptic regions was also observed for CRH1 prepropeptide, to lesser extent for UCN1 prepropeptide, but not for CRH2 prepropeptide (**Figure S3**).

### Comparison of CRH Peptides Primary and 3D Structure

In order to get some insights on potential differences in structure-function relationships between CRH paralogous peptides conserved in eel and in salmon, we further examined their aa sequences and 3D structures.

Human CRH1 was predicted to adopt a variable N-terminal loop conformation (low structure confidence) and a long α-helical conformation for the residues 7 to 39 (**Figure 5A**). We found a similar 3D structure for eel and salmon Crh peptides. In the eel, the α-helix was predicted for aa 8-39 for both Crh1a and Crh1b and for aa 9-41 for Crh2. In salmon, Crh1aα and Crh1aβ have both a α-helical structure for the residues 10-42 and 9-43, respectively, and Crh1bα/Crh1bβ (which have exactly the same sequence) have a α-helical structure for 8-39 residues. Like in human CRH1, the N-terminal structure adopts a variable loop conformation in eel and salmon Crh peptides (**Figure 5A**).

The Crh1 or Crh2 peptides in eel and salmon conserved most of the first 7-18 residues involved in human in receptor binding including, the hydrophobic residues (L^8^, L^10^, L^14^, L^15^, V^18^ and L^19^) shaping hydrophobic interactions and the residues (S^7^, D^9^, T^11^ and R^16^) involved in electrostatic interactions stabilizing the bond peptide conformation within the peptide binding pocket (**Figure 5B**) (55–57).

### Tissue Distribution of *crh* Paralogs in the European Eel and the Atlantic Salmon

In order to get more insight into the fate and functional evolution of the 3R- and the 4R-*crh* paralogs, we compared *crh* paralog tissue expression in both eel and salmon.

#### Tissue Distribution in the Eel

**Figure 6:**
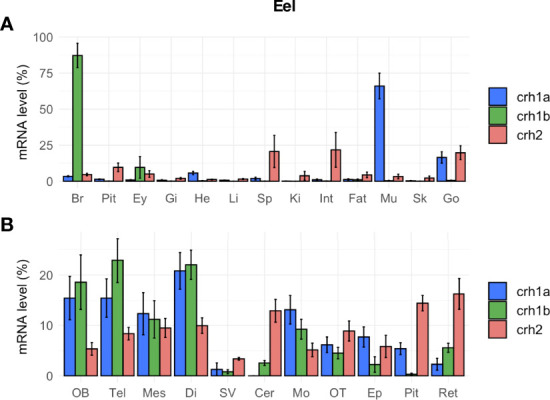
Tissue distribution of *crh* paralogs in silver female European eels as measured by qPCR. **(A)** *crh1a, crh1b and crh2* expression in the brain (Br), pituitary (Pit), eyes (Ey), and various peripheral organs, gills (Gi), heart (He), liver (Li), spleen (Sp), kidney (Ki), intestine (Int), fat (Fat), muscle (Mu), skin (Sk) and gonad (Go). **(B)** *crh1a*, *crh1b* and *crh2* expression in various brain regions: olfactory bulb (OB), telencephalon (Tel), mesencephalon (Mes), diencephalon (Di), saccus vasculosus (SV), cerebellum (Cer), medulla oblongata (Mo), optic tectum (OT), epiphysis (Ep), pituitary (Pit) and retina (Ret). Results (means ± SEM; n=9-10) are presented as percentage per tissue.

In eel, the three *crh* paralogs (*crh1a*, *crh1b* and *crh2)* were not only expressed in the brain but also in other tissues, with differential tissue distribution profiles (**Figure 6A**). All *crh* paralog transcripts were found in the brain, but comparison of their mean cycle quantification (Cq) suggested the following range of expression: *crh1b* (Cq 24.5) > *crh1a* (Cq 26.0) > *crh2* (Cq 29.5). *Crh1a* gene showed the highest expression in the muscle (Cq 23.3), followed by gonads, heart and various other tissues including the brain; its expression in muscle was 15.5-fold than in the brain. In contrast, *crh1b* gene was mainly expressed in the brain while its transcript was also detected at a low level in the eye and at the limit of detection in the other tissues (**Figure 6A**). *Crh2* gene was expressed at a low level in the gonad (Cq 27.3), intestine and spleen, and at lower levels or at the limit of detection in the other tissues. Regarding the distribution of the *crh* paralogs in the brain regions, the two *crh1a* and *b* paralogs showed a similar expression profiles but with much higher levels for *crh1b* than *crh1a* as mentioned above. For both paralogs, higher transcript levels were found in the frontal brain including olfactory bulbs, telencephalon, mesencephalon and diencephalon, and in medulla oblongata as compared to lower levels in optic tectum, cerebellum epiphysis, retina, saccus vasculosus and cerebellum (**Figure 6B**). In the pituitary, *crh1a* was moderately expressed, while *crh1b* levels were at the limit of the detection. *Crh2* transcript was detected in the different brain regions but at very low levels.

#### Tissue Distribution in the Salmon

**Figure 7:**
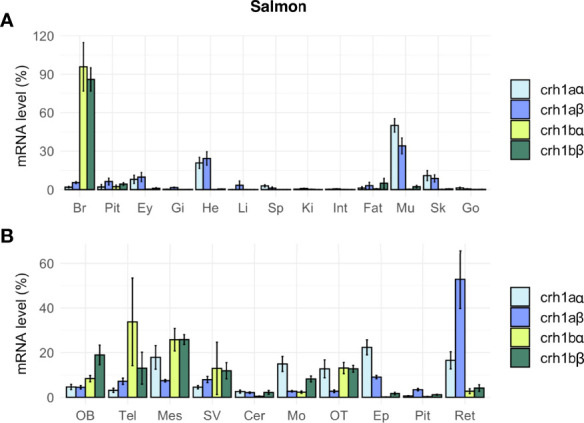
Tissue distribution of crh paralogs in juvenile Atlantic salmon as measured by qPCR. **(A)** *crh1aα*, *crh1aβ*, *crh1bα* and *crh1bβ* expression in the brain (Br), pituitary (Pit), eyes (Ey), aand various peripheral organs, gills (Gi), heart (He), liver (Li), spleen (Sp), kidney (Ki), intestine (Int), fat (Fat), muscle (Mu), skin (Sk) and gonad (Go). **(B)** *crh1aα*, *crh1aβ*, *crh1bα* and *crh1bβ* expression in various brain regions: olfactory bulb (OB), telencephalon (Tel), mesencephalon (Mes), diencephalon (Di), saccus vasculosus (SV), cerebellum (Cer), medulla oblongata (Mo), optic tectum (OT), epiphysis (Ep), pituitary (Pit) and retina (Ret). Results (means ± SEM; n=8-10) are presented as percentage per tissue.

In salmon, the four *crh1* paralogous genes (*crh1aα*, *crh1aβ*, *crh1bα*, *crh1bβ*) were expressed in the brain (**Figure 7A**). Comparison of their mean Cq suggested the following range of expression in the brain: *crh1bβ* (Cq 25.9) > *crh1bα*, (Cq 28.3) > *crh1aα*, (Cq 29.0) > *crh1aβ* (Cq 29.4). The pair of 4R-*crh1aα* and *crh1aβ* paralogs showed a similar tissue distribution profile, with highest expression in muscle [*crh1aα* (Cq 23.8) and *crh1aβ* (Cq 26.7)], followed by heart, then skin, retina, brain and pituitary (**Figure 7A**). The paralogs *crh1aα* and *crh1aβ* showed respectively 13- and 3-fold higher transcript levels in the muscle than in the brain. In contrast, the *crh1b* 4R-pair of paralogs, *crh1bα* and *crh1bβ*, are mainly expressed in the brain with very low or no detectable expression in the other tissues. The paralogs, *crh1bα* and *crh1bβ* showed, respectively 403- and 50-fold higher transcript levels in the brain than in the muscle. When comparing the expression of 4R-pairs of paralogs in various brain regions, some differences were observed between the two *crh1a* paralogs, with the *crh1aβ* mostly expressed in the retina while the *crh1aα* expression more widely distributed (**Figure 7B**).

## Discussion

### Overview of the Evolution of *crh* Family in Vertebrates

The evolution of the CRH neuropeptide family has been previously investigated by other authors, unveiling the existence of five *crh* paralogs in vertebrates, *crh (crh1)*, *crh2*, *ucn (ucn1)*, *ucn2* and *ucn3*, while a single related peptide gene would be present in invertebrate genomes, named in arthropods, diuretic hormone 44 (DH44). The timing of the *crh/ucn* family diversification has been under debate (15, 19, 58). It was first presumed that the whole *crh/ucn* gene family expanded from a chordate *crh/ucn* single ancestor gene, through the two vertebrate WGD (58). However, the comparison of gene chromosome location showed that the *crh/ucn* family evolved in two distinct gene environments, suggesting that the *crh/ucn* family was generated by a first local duplication of a *crh/ucn* ancestor gene, followed by the two successive rounds of vertebrate WGD along with the secondary losses of some paralogs during the rediploidization events (15, 19, 59). Contrary to what the “*crh” versus* “*ucn”* gene names suggest, sequence similarities, together with the location of *crh1*/*crh2*/*ucn1* and *ucn2*/*ucn3* in two distinct gene environments, point out a closer relationship of *ucn* (*ucn1)* with *crh1* and *crh2* than with *ucn2* and *ucn3*. After the local duplication of the common ancestral *crh/ucn* gene, the two vertebrate WGD would have given rise on the one hand to the triplet *crh*, *crh2* and *ucn1*, and on the other hand to *ucn2* and *ucn3* genes (15, 19, 21). Therefore, based on their phylogenetical relationships, and according to the nomenclature recommended for 1R/2R duplicate genes, *ucn (ucn1)* could be named “*crh3”*.

In the present study, we further investigated the evolutionary history of the two *crh* (*crh1* and *crh2*) and their closest paralog *ucn1* throughout vertebrate radiation. Taking advantage of the increasing number of genomes being sequenced, we investigated additional species and key group representatives. *In* our phylogenetical analysis of the prepropeptide amino acid sequences, the three sequences present in the lamprey, a representative of the most basal group of vertebrates (cyclostomes), clustered at the basis of each vertebrate CRH1, CRH2 and UCN1 clades, respectively, supporting the early origin in vertebrates of the triplet *crh1/crh2/ucn1* (19). We also further assessed the presence of the triplet *crh1, crh2* and *ucn1* in the various gnathostome lineages, in agreement with previous studies (15, 21). Among chondrichthyans, in addition to holocephalan (elephant shark) as previously described, we also showed the presence of *crh1, crh2* and *ucn1* in representatives of the two subclasses of elasmobranchs, selacians (catshark), and Batoidae (thorny skate), allowing to generalize the presence of the triplet to all chondrichthyan lineages. Various studies suggest the conservation of CRH role as regulator of the stress axis in chondrichthyans as in other vertebrates [for review (60)]. Regarding CRH2, although it has been first discovered in the holocephan elephant shark, knowledge of its role and mode of action remained to be studied.

In basal sarcopterygians, we took advantage of the recent release of two lungfish giant genomes (61, 62) to search for *crh/ucn* sequences. Thus, in addition to actinistians (coelacanth), where the three genes have been previously reported (15, 21), we also identified the triplet in dipnoans (lungfish). In Australian lungfish we identified full-length *crh2* and *ucn1*, but revealed a pseudogene for *crh1*, while in West African lungfish we were able to retrieve full-length *crh1* and *ucn1* but no *crh2* gene. If these findings do not result from genome assembly artefacts, this suggests species-specific variations in *crh1* and *crh2* conservation or loss among lungfish, with possible compensation of function between *crh1* and *crh2*.

In amphibians, *crh1* and *ucn1* genes, as well as the sauvagine gene, were previously identified while *crh2* gene was reported to have been lost in this lineage (15, 21). Isolated from the skin in the leaf frogs *P. sauvagii* and *Pachymedusa dacnicolor*, sauvagine is considered as a Ucn1 peptide coded by a *ucn1* paralog gene that may have recently highly diverged [for review (63); (64)]. Likely present only in the Phyllomedusinae family, we could not identify any “sauvagine” gene in the available amphibian genomes corresponding to other families. We retrieved only two genes (*crh1* and *ucn1*) in various anuran species (such as *Rana temporaria*) as previously reported for Xenopus. However, in opposite to the previous assumption of the loss of *crh2* in amphibians, we revealed the presence of a *crh2* gene in representatives of caudatan and gymnophionan (such as the axolotl) amphibian lineages. The loss of *crh2* gene would thus be specific to the anuran lineage. In sauropsids, our study further assessed the conservation of the three *crh* genes, *crh1*, *crh2* and *ucn1* in various lineages, including birds, as recently reported (48). In mammals, we found a *crh2* gene only in monotremes and marsupials, confirming its loss in the eutherian lineage (15, 21). These data indicate repeated independent losses of *crh2* in tetrapods, such as in anuran amphibians and eutherian mammals. This opens the way to new investigation on the role and mode of action of *crh2* in order to understand how its function has evolved through the tetrapod radiation.

In actinopterygians, the *crh* gene triplet was previously reported in the spotted gar (21, 22), a representative of holosteans, the sister group of teleosts, and belonging with teleosts to the neopterygians. Our study also showed the conservation of *crh1*, *crh2* and *ucn1* genes in another holostean species, the bowfin. We also identified the triplet in the reedfish, a representative of the basal actinopterygian group of, Polypteriformes. When we looked at chondrosteans, number of copies was doubled for *crh1, crh2* and *ucn1* genes in an Acipenseridae, the sterlet and in a Polyondontidae, the paddlefish. Chondrosteans, both Acipenseridae and Polyondotidae, are known to have experienced lineage-specific WGD (65, 66). In our phylogeny analysis, *crh2* duplicated paralogs clustered together on one side the sterlet paralogs, and on the other side the paddlefish paralogs (**Figure S2**). The *crh2* pairing suggests independent gene doubling events for the *crh2* paralogs in sterlet and paddlefish lineages. Similar relationships have been recently observed for the oxytocin and vasotocin receptors (67) and is in agreement with the independent lineage specific WGD in the Acipenseridae and Polyondotidae (65, 67, 68). Surprisingly, *crh1* and *ucn1* sterlet duplicated paralogs clustered with one of the respective paddlefish paralogs, with well supported nodes, suggesting the duplication events that produced the *crh1* and *ucn1* doubling in chondrosteans may have preceded the split between paddlefish and acipenser lineages. Further investigation may clarify the pattern of the impact of lineage-specific WGD on the fate of *crh1/crh2/ucn1* triplet on chondrosteans, including also investigation of other sturgeon species which displayed additional WGD events.

### Impact of Teleost-Specific WGD and Gene Losses on *crh1* Repertoire

We investigated, by phylogeny and synteny analyses, the impact of the teleost-specific WGD (3R) that occurred early in the teleost lineage, and of the additional WGD (4R) that occurred in some specific groups, as well as of gene losses, on *crh1* repertoire in various extant teleost species. We found that *crh1* duplicated paralogs (*crh1a* and *crh1b*) are present in basal teleosts, elopomorphs (eel and tarpon), osteoglossomorphs (arowana) as well as in many clupeocephalan representatives. Synteny analysis supported the 3R WGD origin of *crh1a* and *crh1b* paralogs in all teleosts. After 3R, both *crh1a* and *crh1b* paralogs have been conserved in most teleost species studied, with a few exceptions where *crh1a* was missing, such as in a tetraodontiform, the fugu, in agreement with a previous report (22). Our observations further suggest that *crh1a* may have also been lost independently in some other teleost species, such as in some clupeiforms, with *crh1a* being present in some Denticipitidae (dendicle herring) and Clupeidae (Atlantic herring, American shad) but detected as a pseudogene in an Engraulidae (anchovy) and missing in some other Clupeidae (pilchard). *Crh1a* was also missing in a gymnotiform (electric eel). Further studies are needed to assess if the absence *crh1a* in these species is related to a loss of function or just a genome sequencing artefact. The conservation of *crh1b* paralog in all teleosts compared to the loss of the *crh1a* paralog in some species, suggests that the *crh1b* paralog is subjected to a higher evolutionary pressure than the *crh1a* duplicate across teleost radiation.

The *crh1a* and *crh1b* paralogs were further duplicated in the polyploid carp (*Cyprinus carpio*) and barbel (*Sinocyclocheilus grahami*) while the other ostariophysians had only a single copy of each paralog. The doubling pairs of paralogs of carp and barbel showed orthologous relationships (**Figure S2**) suggesting duplicated pairs arose from the specific cyprinid WGD allotetraploidization event (CC4R) that occurred after 23 Mya and before the split between *Sinocyclocheilus* and *Cyprinus* lineages (9.7 Mya) (69). Our phylogenetic and syntenic analyses indicated that the four Atlantic salmon *crh1* paralogs paired with the four of other salmonid species, were located on ohnologous genomic regions, in agreement with the 4R-origin of salmonid-specific *crh1a* and *crh1b* paralog doubling (22, 23). In the present study, we named the four salmonid paralogs *crh1aα, crh1aβ, crh1bα* and *crh1bβ*, respectively, in agreement with the nomenclature for salmonid 4R duplicated genes (31).

In contrast to the conservation of 3R-duplicated *crh1a* and *crh1b* paralogs in most teleost species, we found a single *ucn1* paralog type in all teleosts investigated, in agreement with previous reports (15, 21, 70). Synteny analysis showed that the same *ucn1* paralog was conserved in the different teleost species, supporting an early loss after 3R of the other *ucn1* paralog. Two *ucn1* paralogs were found in polyploid cyprinids, and in salmonids. Synteny analysis further supported that the single *ucn1* gene was inherited by salmonid ancestor and duplicated by the 4R.

### Presence and Fate of *crh2* in Teleosts

The absence of *crh2* gene in teleosts was assumed to result from the loss of *crh2* gene in a teleost ancestor prior the 3R WGD (15, 19, 21, 22). In order to trace the evolutionary trajectory of the *crh2* gene in teleosts, we investigated a large number of species from various teleost groups. Strikingly, our gene search and phylogenetic analysis revealed the presence of a *crh2* gene in representative species of basal teleost taxa, elopomorphs and osteoglossomorphs, as well as of some basal clupeocephalan orders such as Clupeiformes, Gonorynchiformes, Characiformes and Gymnotiformes. In contrast, *crh2* gene was missing in Siluriformes and Cypriniformes as well as in all euteleosts investigated.

In teleost species where a *crh2* was present, a single gene was identified. The presence of only a single *crh2* gathering in one clade for teleost species in the phylogenetic analysis, may have suggested an early loss of one of the 3R-duplicated paralogs before the teleost radiation. Synteny analysis confirmed that *crh2* gene environment has been duplicated in accordance with the 3R WGD event. However, gene neighborhood exploration revealed that the *crh2* gene was not located on the same paralogon in osteoglossopmorphs, as compared to elopomorphs and clupeocephalans, and corresponded to different 3R-paralogs. This raises the question about the timing of *crh2* 3R-duplicate gene loss. According to our synteny study, both 3R-*crh2* paralogs would have been retained after the divergence of the teleosts, with elopomorphs and clupeocephalans having conserved one 3R *crh2* paralog, that we named *crh2a* and lost the *crh2b* paralog, while osteoglossomorphs having conserved the *crh2b* paralog and lost the *crh2a* paralog. The lack of *crh2* gene in cypriniforms and siluriforms indicates in addition to the loss of *crh1b* in a clupocephalan ancestor, *crh2a* paralog was lost independently twice in the ostariophysian lineage, one time before the radiation of cypriniforms and the other time before the radiation of siluriforms. We could not find any *crh2* genes either in any representatives of basal euteleostean taxa, including esociforms, salmoniforms, galaxiiforms and osmeroiforms, supporting the loss of *crh2b* paralog before the radiation of clupeocepalans, followed by the loss of *crh2a* paralog before the radiation of euteleosts.

The present data allow us to refute the previous assumption of the absence of *crh2* in teleosts and to infer the evolutionary scenario of *crh2* in teleosts. *Crh2*, present in basal actinopterygian taxa, polypterids, chondrosteans, and holosteans, would have been inherited by teleost ancestor and duplicated *via* the teleost-specific 3R WGD into *crh2a* and *crh2b* paralogs. The *crh2a* paralog would have been lost in osteoglossomorphs while it would have been conserved in elopomorphs and clupeocephalans. Conversely, *crh2b* would have been conserved in osteoglossomorphs but lost independently in elopomorphs and clupeocephalans. While *crh2a* has been conserved in various basal clupeocephalan taxa (Clupeiformes, Gonorynchiformes, Characiformes and Gymnotiformes), it would have been lost independently in some other basal clupeocephalan taxa (Siluriformes, Cypriniformes), as well in the euteleostean lineage leading to the lack of any *crh2* in the majority of extant teleost species.

### Conservation of Structure-Function Relationships of CRH Peptides

The vertebrate CRH1 and CRH2 precursors have all the same overall structure, including a signal peptide, a cryptic region with at its C-terminus a proteolytic cleavage site and the C-terminal region coding for the CRH peptide with a C-terminal Gly-Lys amidation site. The different branch sizes of CRH prepropeptides in our inferred phylogenetic tree indicates different selective constraints between the prepro-CRH. Together with the sequence alignment it showed a higher variability of CRH2 than CRH1 prepropeptide sequences among vertebrates in agreement with previous observations (15, 21). This suggests a higher evolutionary constraint on CRH1 than CRH2, which is also supported by the conservation of *crh1* gene in all vertebrates, while *crh2* gene had been repeatedly lost in various lineages, such as in anuran amphibians, eutherian mammals, and some teleost taxa. The strong conservation of CRH1 has been associated to the evolutionary pressure related to its central role as coordinator of the corticotropic axis in all vertebrates, while the potential physiological roles of CRH2 have still to be investigated.

When comparing the sequences of Crh1a, Crh1b and Crh2 peptides themselves, a striking sequence conservation is observed, as for example seen in eel and salmon. In salmon, the 4R-duplicated Crh1bα and bβ peptides even have exactly the same sequence. 3D structure prediction revealed, as for human CRH1, the conservation of the alpha helical structure in eel and salmon Crh peptides including eel Crh2. The action of the neuropeptides of CRH family, including CRH1 and CRH2, UCN1 as well as UCN2 and UCN3, is mediated by receptors belonging to the class B of G protein-coupled receptors (GPCR) of the secretin-like receptor superfamily [ (71, 72); for review (73)]. Two CRH receptors, CRHR1 and CRHR2 arose from the vertebrate WGD and have been first characterized in mammals (71, 74–78). The recent single-particle cryoelectron microscopy (cryo-EM) resolution of CRH1 and UCN1 complexes to the CRH receptors revealed a conserved mode of ligand binding and receptor activation for the CRH family peptides and their receptors (55, 56). CRH binding to the receptor induces the rearrangement of the receptor transmembrane domains driving the receptor activation and the coupling with G proteins (55). The C-terminal helical segment of CRH peptide interacts with the receptor extracellular loops promoting recognition specificity and the N-terminal helix segment that bind to the receptor transmembrane domain core pocket stimulates the receptor activation and G protein coupling (55, 57). In addition to the helical structure conservation, Crh peptides in both salmon and eel conserved the residues reported in human CRH as the key residues for the receptor recognition and activation. These data suggest that all these peptides conserved CRH biological ability to stimulate CRH receptors. CRH role has been investigated in teleosts using heterologous CRH1 peptides *in vitro* and *in vivo*. In eel, bovine CRH is active and able to stimulate *in vitro* release of growth hormone (GH) by pituitary cells (79). In salmon, ovine CRH stimulates thyroid-stimulating hormone (TSH) release (80), as well as transcription of *gh* and *tshβ* subunit paralogs (81) *in vitro*. Central administration of ovine or of rat/human CRH increases locomotor activity in Chinook salmon (*Oncorhynchus tshawytscha*) (82) and downstream migration in smolt coho salmon (*Oncorhynchus kisutch*) (83).

While CRH1 peptide in mammals is known to exert mainly its actions through the activation of the CRHR1 receptor for which it shows higher affinity, little is known about CRH2 receptor preference. The functional characterization of chicken (*Gallus gallus*) CRH2 using recombinant chicken CRHR1 and CRHR2 expressed in CHO cells revealed CRH2 is more selective for CRHR2 than CRHR1 while CRH1 activates both receptors with similar potency (48).

Gene coding for CRH receptors (*crhr1* and *crhr2)* underwent duplication during the teleost 3R WGD, but one of the 3R-*crhr2* paralog would have been early lost in the teleost lineage. Some species have conserved the two 3R-paralogs of *crhr1* such as the tilapia (*Oreochromis niloticus*) (77). Japanese medaka (*Oryzias latipes)*, belongs to the species that have conserved the pair of 3R-*crh1* paralogs, but a single paralog of Crha and Crhb (70). Both medaka Crh1a and Crh1b peptides were reported to be able to stimulate the two CRH receptor types (Crhr1 and Crhr2) transiently expressed in HEK293T cells and subsequently cAMP production with an EC50 for Crh1b peptide slightly lower for both receptors (70). Eels have conserved 3 genes coding for CRHR, two *crhr1* and a single *crhr2* (LOC118216809, LOC118218028 and LOC118234689). Atlantic salmon has two pair for both *crhr1* (LOC106600876 and LOC105023366) and *crhr2* (LOC106569328 and LOC106599903), each duplicate arising likely from the duplication of a single 3R-paralog *crhr1* and *crhr2* during the salmonid tetraploidization (4R). Crhr1 and Crhr2 in chum salmon (*Oncorhynchus keta*) show highly conserved amino acid sequences with human orthologs (84). Functional study of these receptors showed that rat/human CRH can bind and activate both receptor type with similar potency (84). Further functional experiments using homologous CRH peptides and CRHR will contribute to deeper understanding of the functional evolution of the multiple CRH-CRHR systems in fish.

### Potential Major Role of *crh1b* Paralogs in the Brain in Eel and Salmon

Conservation of paralogous gene after duplication is under selective pressure likely related to either amplification of function, functional partitioning (subfunctionalization), or emergence of new function (neofunctionalization). The strong conservation of CRH1 and CRH2 peptide sequences and 3D structure as discussed above suggests that conservation of multiple *crh* genes is not related to major differences in the abilities of the peptides to bind and activate CRH receptors. Selection may have rather targeted differential *crh* gene tissue expression and regulation. In the present study we got some insights on the potential differential functions of *crh* paralogous genes by comparing their tissue expression in two key models, a basal teleost, an elopomorph, the European eel, which has conserved both 3R-*crh1a* and *crh1b* paralogs, as well as a single *crh2* gene, and a salmonid, the Atlantic salmon, which possesses four *crh1* paralogs resulting from 4R duplication of *crh1a* and *crh1b*, and no *crh2* gene. Furthermore, in addition to the relevance of their phylogenetical position and *crh* repertoire among teleosts, eel and salmon are also of wide interest in biology, ecology, conservation or aquaculture. It should be noted that our study was performed on juvenile fish and that the relative tissue expression of *crh* paralogs may vary according to maturation status and other physiological conditions, such as stress.

All *crh* paralog transcripts could be detected by qPCR in the brain, in the eel and salmon, including in the brain region (mesencephalon/diencephalon) where hypophysiotropic neurons are located. Early immunocytochemical studies in mammals located CRH neurons in the paraventricular nucleus of the hypothalamus (11). Since then, this principal center of CRH expression, with neurons projecting to the median eminence, has been confirmed in other tetrapods [birds: (85, 86); reptiles: (87); amphibians: (88–90). In teleosts, due to the absence of hypophyseal portal system and the direct innervation of the pituitary by hypophysiotropic neurons, the situation is slightly different, with CRH neurons in the preoptic area projecting up to the pituitary [*Carassius auratus* and *C. carpio*: (91); white sucker *C. commersoni*: (92, 93); *Anguilla* species: (94); *Salmo*, *Oncorhynchus* and *Anguilla* species, *Mugil ramada* and *Myoxocephalus octodecimspinosus*: (95); rainbow trout: (96); Chinook salmon: (97); tilapia *Oreochromis mossambicus*: (98)].

In our study, the detection in eel and salmon of the expression of each *crh* paralog in the brain hypophysiotropic region, suggests that all *crh* paralogs may potentially be involved in the major role of CRH as brain regulator of the corticotropic axis. However, comparison of Cq values indicates that *crh1b* paralog is expressed at a higher levels than *crh1a* in the brain in the eel, and a similar situation is observed in the salmon for the 4R-pair of *crh1b* paralogs as compared to the 4R-pair of *crh1a* paralogs. Furthermore, comparison of the distribution of each paralog in the brain and various peripheral tissues showed that *crh1b* paralogs (*crh1b* in the eel and the pair of 4R-*crh1b* in salmon) are mostly expressed in the brain, while *crh1a* paralogs (*crh1a* in eel and the pair of 4R-*crh1a* in salmon) are mostly expressed in the muscle. Our results are in accordance with the distribution of the 3R-*crh1* paralogs in other teleosts (22; 70). In medaka *crh1a* is more expressed in the muscle, heart and gonad than in the brain. In the brain, c*rh1b* is broadly expressed including in the hypophysiotropic neurons in zebrafish, in *Astatolipia burtoni* and medaka while *crh1a* paralogs are found in different location through the brain including the ventral hypothalamus but not expressed in the hypophysiotropic neurones (22; 70).

This suggests an early functional partitioning between *crh1* paralogs issued from the 3R before teleost radiation, with *crh1b* playing a major role in the brain and *crh1a* in the muscle. The potential major role of *crh1b* paralog in the neuroendocrine control of the corticotropic action, may represent the evolutionary constraint that led to the conservation of *crh1b* paralog in all extant teleost species while *crh1a* paralog has been lost in some taxa/species such as in tetraodontiforms (fugu) and some clupeiforms and gymnotiforms.

Concerning 4R-pairs of *crh1a* and *crh1b* paralogs in salmon, their general central and peripheral tissue distribution is quite similar within each pair, reflecting some low differentiation of 4R-issued paralogs. Some differences could still be observed when comparing the expression of salmon 4R-paralogs between various brain regions, as also previously reported (23). The authors showed that *crh1bα* (in their article *crf1b1)* was the most abundant in the post-smolt brain but that the four paralogs may be involved in the response to various stress exposures (hypoxia, chasing, combination of both and confinement) (23). *Crh1bα* (in their article *crf*-*b1*) was also suggested to be involved in the activation of the pituitary interrenal axis leading to the elevation of cortisol at smoltification (53).

Apart from the hypothalamus, a widespread distribution of CRH peptides or transcripts in the brain has been reported in other vertebrates [mammal: (99); bird: (100); reptile: (87); amphibian: (89)]. In our study, the different paralogs characterized in the European eel and the Atlantic salmon are expressed in the various brain regions. This supports that in addition to be the activator of neuroendocrine axes, CRH1 is involved in the control of many other brain physiological functions including various behaviors [for review: (10)] such as food intake and feeding behavior [mammals: (101); teleosts: (29, 102)], sensory processing, locomotion and migration [vertebrates: (103); teleosts: (29)].

### Potential Major Autocrine/Paracrine Role of *crh1a* Paralogs in Muscle and Heart in Eel and Salmon

CRH1, originally isolated from the hypothalamus for its neuroendocrine role on the pituitary, is also secreted locally in various peripheral tissues, where it can exert autocrine or paracrine effects, as its receptors are widely distributed [for review: (10)].

Strikingly, the highest level of expression for eel *crh1a* and salmon 4R-*crh1a* pair was observed in the skeletal muscle, as compared to other tissues including the brain and various peripheral tissues. A relatively high expression of *crh1a* was also seen in the heart in salmon and eel. In contrast, *crh1b* paralog transcripts were at low levels in the skeletal muscle in the eel and undetectable in the salmon, and *crh1b* paralogs were not detected in the heart in both species. Mirroring the potential major role of *crh1b* paralog in the brain, this suggests a major potential role of *crh1a* paralog in the control of muscle and heart functions in teleosts, further supporting the functional partitioning between 3R-*crh1* paralogs.

The expression of *crh1* in skeletal and cardiac muscles has already been reported in other teleosts. In *A. burtoni, crh1b* expression was detected using RT-PCR in skeletal muscle and heart (104), but these results were not confirmed for heart and not tested for muscle by qPCR (59). *Crh1b* was also detected by qPCR in muscle and heart of *Schizothorax prenanti* at low and middle levels, respectively (105), and of *Schizothorax davidi* at low levels (106). In the medaka, high levels of *crh1a* transcripts have been reported in muscle and heart (70). In zebrafish, both *crh1a* and *crh1b* mRNAs were detected in the heart (107). These data suggest possible species-specific variations in the functional partitioning and the relative roles of *crh1a* and *crh1b* paralogs in skeletal muscle and heart, according to teleost species.

Expression of *crh1* has also been reported in the heart of *Xenopus laevis*, but was absent in the muscle (108). No matter which paralogs are involved, the expression of *crh1* in the heart in teleosts allows to raise the hypothesis that CRH1 may have a protective role against stressors in the heart, as shown for CRH-related peptides in mammals [for review: (109)]. In zebrafish, it has recently been reported that hypoxia-reperfusion exposure increased cardiac *crh1b* expression, and rat/human CRH was protective against hypoxia/reoxygenation-induced apoptosis *in vitro* in this tissue (107). These data suggest a potential direct action of CRH-like peptides on cardiac myocytes without the involvement of nervous system. CRH in teleosts may also modulate glucose uptake and insulin sensitivity, as urocortin 2 does in mouse skeletal muscle (110). Finally, it can be hypothesized that CRH1 modulation of locomotor and migratory activities in teleosts, as shown in salmon species [Chinook salmon: (82, 111); chum salmon: (83); coho salmon: (112)], may be mediated not only *via* central actions on behavior and neuroendocrine axes, but also *via* direct peripheral actions on heart and muscle function. Injection of CRH1 in hypophysectomized rats increases locomotor activity demonstrating that CRH1 can produce behavioral activation independently of its effect on the corticotropic axis (113). In salmon and eel, CRH1 effects in the brain would be mostly ensured by *crh1b* paralogs while the effects on skeletal muscle and heart by *crh1a* paralogs.

### Potential Autocrine/Paracrine Roles of *crh1* Paralogs in Various Other Organs in Eel and Salmon

In the eel, after muscle and heart, *crh1a* paralog was found to be expressed in the gonads (ovarian tissue from prepubertal silver eels). Low expression of c*rh1a* 4R-paralogs could also be detected in ovarian tissues in immature smolt salmons. In contrast, *crh1b* paralog transcripts were not detectable in the gonads of both species. Expression of *crh1* has been previously reported in the ovary or testis of some other teleosts, such as common carp (114), *A. burtoni* [*crh1b*: (104)], fathead minnow (*Pimephales promelas*) (115). A recent study in zebrafish showed that both *crhα* and *crhβ* paralogs (corresponding to *crh1*a and *crh1b*) are expressed in the ovary, with a differential regulation of their expression according to vitellogenic stages (116). Furthermore, the authors demonstrated an inhibitory effect of CRH1 on estradiol production by zebrafish follicular cells *in vitro*. In mammals, CRH1 immunoreactivity or expression was reported in gonads, testis [rat: (117–119)] and ovary [rat: (120); human: (121, 122)]. In the testis, CRH1, produced by the Leydig cells of the testis, exerts autocrine inhibition of testosterone biosynthesis (118, 123). Similarly, in the ovary, CRH1 inhibits steroid biosynthesis as shown *in vitro* [rat and human granulosa cells: (124); human granulosa-lutein cells: (125); human thecal cells: (126); mouse preantral follicles: (127)]. Altogether these data suggest a conserved direct inhibitory role of CRH1 on gonadal steroidogenesis in vertebrates, which may participate, together with interactions between neuroendocrine axes, in the well-known stress-related impairment of reproduction, as observed in many species [for review (128)]. In teleosts, this role of CRH1 on the gonads may likely be fulfilled by either one or the other *crh1* paralog according to species.

In the pituitary, we detected a low expression of 3R-paralog *crh1a* in eel and of 4R-paralog *crh1aβ* in salmon. This is in agreement with the recent report of the expression of “*crf1a2*” (corresponding to *crh1aβ* in our study) in the salmon pituitary (23). In other teleosts, the expression of *crh* in the pituitary has been mostly studied for *crh1b*, the firstly identified “classical” *crh*, and absence [goldfish, Northern-blot: (129); European flounder (*Platichthys flesus)*, RT-PCR: (130); *A. burtoni* RT-PCR: (104)] or low expression [*S. prenanti*, qPCR: (105); grass carp (*Ctenopharyngodon idellus*), RNAseq: (131); RT-PCR: (59)] were reported. When *crh1a* was studied, no expression could be detected in the pituitary [medaka, ISH: (70); grass carp, RNAseq: (131); *A. burtoni* RT-PCR: (59)]. Together with ours, these data suggest species-specific variation in the expression of 3R-*crh1a* or *b* paralog in the pituitary across teleost species. In other vertebrates, the amphibian *Xenopus laevis* (108) and various mammals [rat: (132); baboon: (133)], CRH expression (transcript or peptide) have been reported in the pituitary. Altogether, this indicates that CRH may exert a paracrine/autocrine role in the pituitary in addition to its neuroendocrine major role, in teleosts as in other vertebrates.

In the retina, we detected the expression of *crh1a* in the eel while the *crh1b* was at the limit of the detection. In the salmon, the 4R-*crh1aβ* was much more expressed than the other 4R-*crh1* paralogs. The relatively higher expression of a *crh1a* paralog in both eel and salmon as compare to *crh1b* reflects a functional differentiation of the 3R paralogs in these species. The low retinal expression levels of 4R-*crh1aα* as compared to 4R-*crh1aβ* indicates that functional differentiation also occurred between the 4R-*crh1a* paralogs. Early immunocytochemical studies reported the presence of CRH in the retina in teleosts [goldfish: (134)] as well as in birds [chicken: (135)], reptiles [turtle: (136)] and mammals [rat: (137, 138)]. Among teleosts, when retina was tested, it was shown to be a site of expression of *crh1* [*crh1a* in medaka: (70); *crh1b* in *Shizothorax* species: (105, 106); *crh1a* and *crh1b* in *A. burtoni*: (59, 104)]. Using ISH, 22 observed some differences in the retinal expression of *crh1 a* and *b* paralogs between two teleosts, *A. burtoni* and zebrafish (22). *Crh1a* expression was either absent (zebrafish) or present (*A. burtoni*) in the retina, while *crh1b* expression was present but in distinct cells: amacrine and ganglion cells in zebrafish; amacrine and bipolar cells in *A. burtoni.* As for other organs, this suggests species-specific variations among teleosts in the respective roles of *crh1a* and *b* paralogs in the retina. Whatever the *crh1* paralog involved, the expression of CRH in the retina supports possible local autocrine/paracrine actions of CRH1 in the neuromodulation of retinal function in teleosts (22).

### Potential Pleiotropic Roles of *crh2* in the Eel

This study is the first one to demonstrate the presence of *crh2* gene in teleosts and thus to investigate the tissue distribution of its expression in a teleost species. Measure of *crh2* transcripts by qPCR in the eel revealed a tissue expression profile distinct from those of *crh1a* and *crh1b*. *Crh2* expression was low and widely distributed in central and peripheral tissues, with no striking major site of expression. The general tissue distribution shows that, differently from *crh1a* and *b*, the main sites of expression of *crh2* are the gonads, the intestine and the spleen. However, even in these tissues, *crh2* transcripts were at lower (gonads, spleen) or similar (intestine) levels than those of *crh1a*, as suggested by Cq comparison, while *crh1b* transcripts were at the limit of detection. CRH activation of the corticotropic axis, in response to stress, is known to stimulate digestive tract motility as for instance in human (139). The expression of *crh2* and *crh1a* in the eel intestine suggests an additional autocrine/paracrine action of CRH in addition to its central effect. The expression of *crh2* and *crh1a* in the spleen in the eel also suggests a direct action of CRH on immune function. Chronic stress is recognized to impair immunity and notably humoral response in mammals, an effect involving not only CRH-activated corticotropic axis but also a direct effect of CRH on immune cells as indicated by the expression of CRH receptors by splenic B cells (140). *Crh2* expression in the digestive tract and spleen of chicken has also been reported (48). When looking at the detailed central distribution, *crh2* expression was very low, with slightly higher transcript levels in the retina, pituitary and cerebellum than in the other brain parts, a distribution profile also different from those of *crh1a* and *b*.

The low and wide expression of *crh2* in the eel does not support any major specific function for *crh2*, as compared to *crh1a* and *crh1b* which in contrast may play out predominant roles in the muscle and in the brain, respectively. To the best of our knowledge only one other study investigated the central and peripheral tissue distribution of *crh2* in vertebrates: in the chicken, qPCR analysis showed that *crh2* is widely expressed in various brain regions as well in multiple peripheral tissues (48), similarly to our finding in the eel. Differently, a very limited distribution of *crh2* was observed in the spotted gar, but using ISH on the brain, with *crh2* expression restricted to a specific nucleus of the hindbrain and in particular no expression detected in the retina. Further studies, including representatives of other actinopterygians as well as of other vertebrate taxa having conserved *crh2*, are clearly needed to evaluate common and divergent patterns of *crh2* expression across vertebrate radiation.

The low expression and wide distribution pattern of *crh2* in a basal teleost, the eel, suggests a low specific evolutionary constraint, paving the way to the repeated losses of *crh2* through teleost radiation, such as shown in our study for siluriforms, cypriniforms and the euteleostean lineage, leading to the absence of *crh2* in the majority of extant teleost species. A similar situation may have occurred in tetrapods with the losses of *crh2* in anuran amphibians and eutherian mammals.

## Conclusion

The pituitary gland and its control by brain neurohormones is a major anatomical and functional innovation of vertebrates. Families of neuropeptides and receptors, pre-existing in metazoans before the emergence of vertebrates, have been diversified *via* the two vertebrate WGD and recruited for the control of the pituitary (for review: 24). This is the case for CRH, the neurohormone responsible for the control of the vertebrate corticotropic axis, which represents a typical case of neofunctionalization in the CRH/UCN family, as compared to the ancestral role in non-vertebrate metazoans. The present study further assesses the role of vertebrate *crh1* paralog in the neuroendocrine control of the corticotropic axis, a strong evolutionary constrain that led to its conservation in all extant vertebrates. It highlights the subfunctionalization between the duplicated *crh1* paralogs issued from teleost-specific WGD (3R), with *crh1b* assuming the neuroendocrine control of the pituitary and being conserved in all extant teleost species, while *crh1a* being mainly involved in local autocrine/paracrine functions, with a lower selective pressure leading to species-specific variations in *crh1a* expression and functions, up to the loss of *crh1a* paralog in some teleost species. Concerning the more recently identified vertebrate *crh2* paralog, this study reveals some wider conservation across vertebrates than previously assumed, with its presence in additional vertebrate groups including elasmobranchs, dipnoans, caudatan and gymnophionan amphibians, actinopterygian polypterids, chondrosteans and teleosts. The low and wide tissue expression of *crh2* as observed in a basal teleost, the eel, suggests various local autocrine/paracrine functions. Future studies should aim at investigating the regulation of *crh2* expression in relation to development, maturation and stress challenges in teleost species, such as the eel, that have retained this paralog. These results revisit the repertoire of *crh* in teleosts and highlight functional divergences that may have contributed to the conservation of various *crh* paralogs in teleosts. This study also supports that no major specific function of *crh2* paralog would have led to low evolutionary constraint and repeated losses of *crh2* across vertebrate radiation, such as in some amphibians, in eutherian mammals and in various teleosts including the large group of euteleosts.

## Data Availability Statement

All relevant data is contained within the article: The original contributions presented in the study are included in the article/**Supplementary Material**. Further inquiries can be directed to the corresponding author.

## Ethics Statement

The animal study was reviewed and approved by Cuvier Ethic Committee France (No. 68–027).

## Author Contributions

Design of the study: GM, PM, KR, SD. Experiments: GM, XM. Analysis and interpretation of data: GM, SA, KR, SD. Writing of the manuscript: GM, KR, SD. All the authors reviewed and approved the submitted version of the manuscript.

## Funding

This work was supported by grants from Alliance Sorbonne University DESYNCHRO SU-14-R-CDV-07-1 and from the French National Research Agency SALTEMP No ANR-12-ADAP-0021 and NEMO No. ANR- 14-CE02-0020.

## Conflict of Interest

The authors declare that the research was conducted in the absence of any commercial or financial relationships that could be construed as a potential conflict of interest.

## Publisher’s Note

All claims expressed in this article are solely those of the authors and do not necessarily represent those of their affiliated organizations, or those of the publisher, the editors and the reviewers. Any product that may be evaluated in this article, or claim that may be made by its manufacturer, is not guaranteed or endorsed by the publisher.

## Supplementary Material

Supplementary Table 1 References of crh neuropeptide sequences used for phylogenetic analysis. The table provides the species names, gene prediction/annotation, names, identifiers, and locus references.

Supplementary Table 2 References and location of the genes used for the crh neuropeptide synteny analysis. The table lists the neighboring genes of the *crh* genes in reedfish, eel, arowana, herring, zebrafish, pike, salmon. The data includes gene identifiers, and location.

Supplementary Table 3 Primer sets used for quantitative PCR. The table provides the name and 5’-3’ nucleotide sequences of the primer sets used for qPCR of the *crh1* and *crh2* paralog genes and gene references in European eel and Atlantic salmon.

Supplementary Figure 1 Venn diagram showing the distribution of the gene family members on the crh-bearing chromosomes in the reedfish genomes.

Supplementary Figure 2 Maximum-likelihood phylogenetic tree of CRH precursor amino acid sequences with a focus in actinopterygian representatives. Maximum-likelihood phylogenetic tree of CRH precursor amino acid sequences with a focus in actinopterygian representatives. Phylogenetic relationships of the prepro-CRH was inferred using the PhyML algorithm with the WAG substitution matrix and the best nearest neighbour interchange (NNI) and Subtree Pruning and Regrafting (SPR) improvement algorithm. Numbers at the node indicate the confidence percent of 100 bootstrap replication. The three gnathostome monophyletic clades are indicated with different branch colors, purple for the CRH1, pink for the CRH2 and green for the UCN1. Taxonomic group names are indicated at the right of the tree, with colors and the corresponding animal silhouettes (http://www.phylopic.org).

Supplementary Figure 3 Sequence logo of CRH1, CRH2 and UCN1 prepropeptides. The logo sequence was built for each CRH prepropeptides from the sequence alignment of the phylogenetic tree (**Figure 1**) using CLC Main Workbench (QIAGEN). Consensus position of different domains in the CRH prepropeptides are underlined: the signal peptide, the cryptic region, the CRH peptides cleavage site (indicated by an arrow) and the CRH peptides with its amidation site. It shows a high conservation of CRH1, CRH2 and UCN1 peptide sequences and a good conservation of the peptide signal. Conserved motifs are observed within the cryptic of CRH1 and UCN1 precursors but not for CRH2 precursors that show high sequence divergences within the cryptic regions. CRH1 precursor sequences of mammals and sauropsids are longer than other CRH1 sequences (around 30 aa longer), the additional residues are distributed between the conserved motifs of cryptic region.
