# Supplementary figures and images for: New Insights Into the Evolution of Corticotropin-Releasing Hormone Family With a Special Focus on Teleosts
Source: Front Endocrinol (Lausanne). 2022 Jul 22;13:937218. doi: 10.3389/fendo.2022.937218 (PMC9353778; doi:10.3389/fendo.2022.937218)

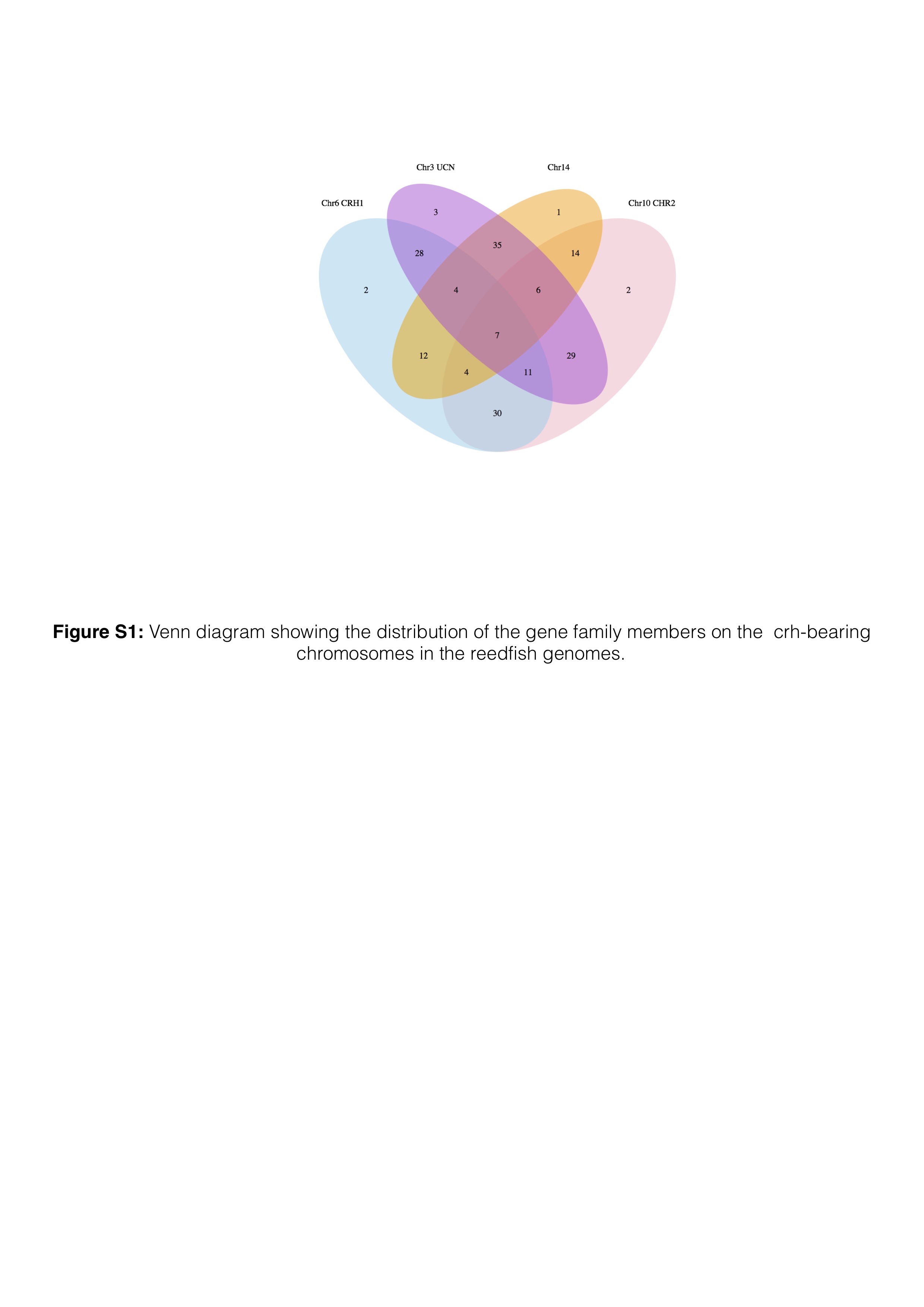

Supplement: Supplementary Figure 1 — Venn diagram showing the distribution of the gene family members on the crh-bearing chromosomes in the reedfish genomes. [file Image_1.jpeg]

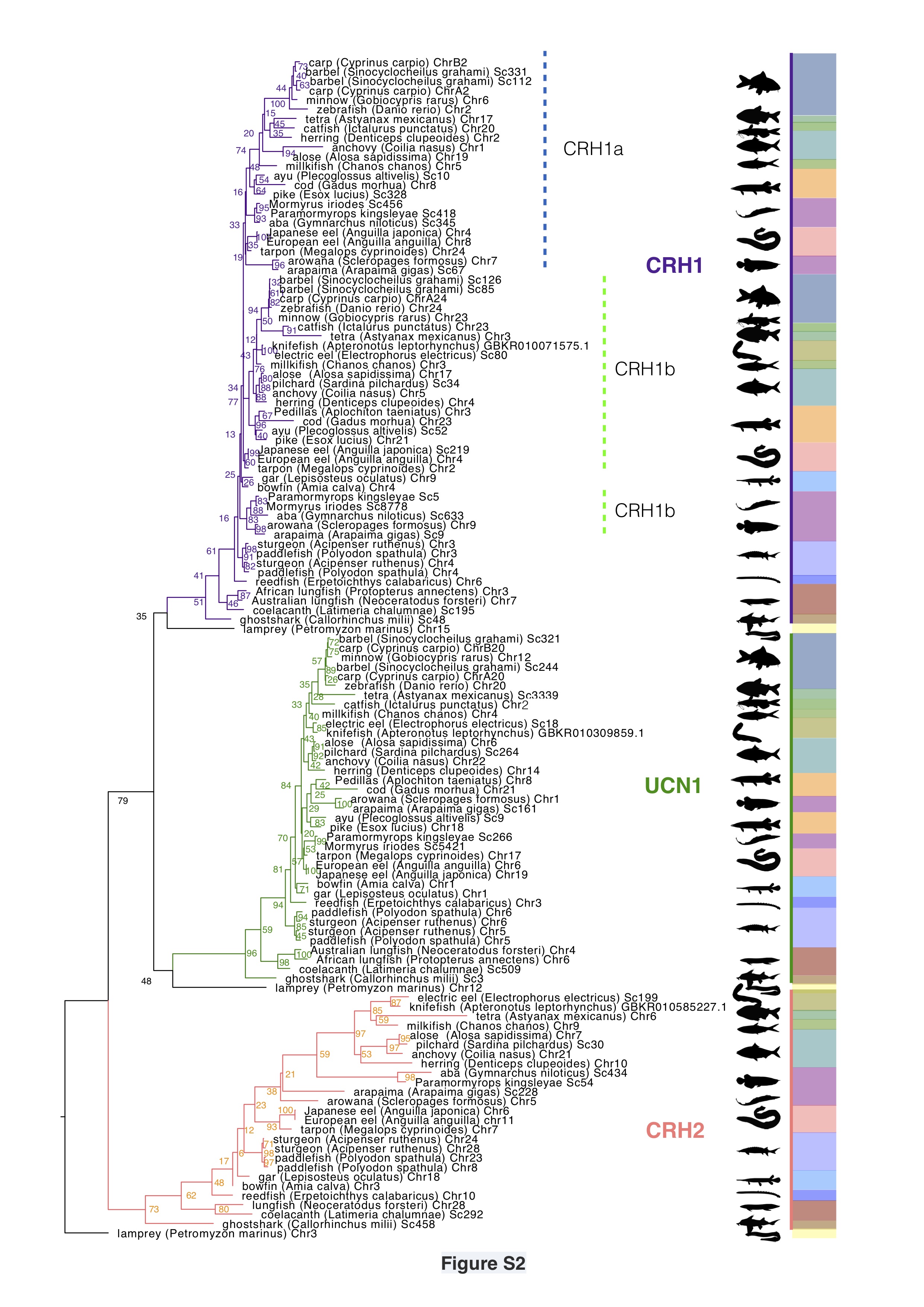

Supplement: Supplementary Figure 2 — Maximum-likelihood phylogenetic tree of CRH precursor amino acid sequences with a focus in actinopterygian representatives. Maximum-likelihood phylogenetic tree of CRH precursor amino acid sequences with a focus in actinopterygian representatives. Phylogenetic relationships of the prepro-CRH was inferred using the PhyML algorithm with the WAG substitution matrix and the best nearest neighbour interchange (NNI) and Subtree Pruning and Regrafting (SPR) improvement algorithm. Numbers at the node indicate the confidence percent of 100 bootstrap replication. The three gnathostome monophyletic clades are indicated with different branch colors, purple for the CRH1, pink for the CRH2 and green for the UCN1. Taxonomic group names are indicated at the right of the tree, with colors and the corresponding animal silhouettes (http://www.phylopic.org). [file Image_2.jpeg]

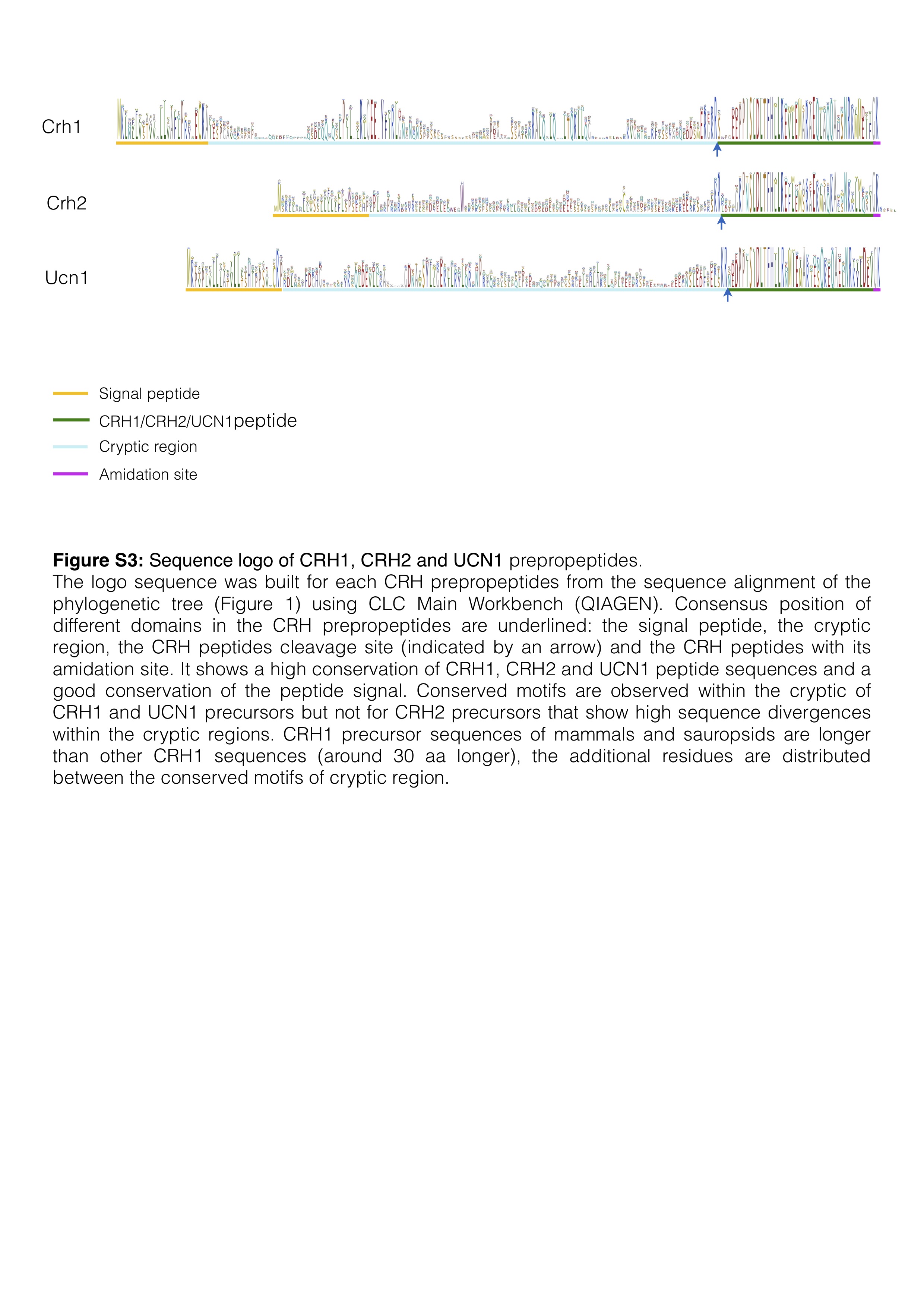

Supplement: Supplementary Figure 3 — Sequence logo of CRH1, CRH2 and UCN1 prepropeptides. The logo sequence was built for each CRH prepropeptides from the sequence alignment of the phylogenetic tree ( Figure 1 ) using CLC Main Workbench (QIAGEN). Consensus position of different domains in the CRH prepropeptides are underlined: the signal peptide, the cryptic region, the CRH peptides cleavage site (indicated by an arrow) and the CRH peptides with its amidation site. It shows a high conservation of CRH1, CRH2 and UCN1 peptide sequences and a good conservation of the peptide signal. Conserved motifs are observed within the cryptic of CRH1 and UCN1 precursors but not for CRH2 precursors that show high sequence divergences within the cryptic regions. CRH1 precursor sequences of mammals and sauropsids are longer than other CRH1 sequences (around 30 aa longer), the additional residues are distributed between the conserved motifs of cryptic region. [file Image_3.jpg]
