# Supplementary material for: New Insights Into the Evolution of Corticotropin-Releasing Hormone Family With a Special Focus on Teleosts
Source: Front Endocrinol (Lausanne). 2022 Jul 22;13:937218. doi: 10.3389/fendo.2022.937218 (PMC9353778; doi:10.3389/fendo.2022.937218)
Supplement: Supplementary file 7 [file DataSheet_1.pdf]

Refeerences of the Supplemental files

Chen, Z., Omori, Y., Koren, S., Shirokiya, T., Kuroda, T., Miyamoto, A., ... & Burgess, S. M. (2019). De novo assembly of the goldfish (Carassius auratus) genome and the evolution of genes after whole-genome duplication. Science advances, 5(6), eaav0547.
